# Comparative Investigations of the Neurophysiological Mechanisms of Escape Behavior: A Course-based Undergraduate Research Experience

**DOI:** 10.59390/001c.160661

**Published:** 2026-06-04

**Authors:** Eric R. Wengert, Roshan A. Jain

**Affiliations:** 1 Bi-College Interdisciplinary Neuroscience Program Haverford College; 2 Department of Medical Education Geisinger College of Health Science; 3 Biology Haverford College

**Keywords:** Mauthner cells, zebrafish, MATLAB, Course-based Undergraduate Research Experience, CURE, escape behavior, neuropharmacology, neurophysiology, giant interneurons, calcium imaging

## Abstract

Incorporating authentic research into undergraduate neurobiology courses significantly enhances student learning and builds the foundation for rigorous neuroscience training. These course-based undergraduate research experiences (CUREs) are high impact: they foster the skills of scientific inquiry, logical reasoning, scientific writing and communication, increase academic performance, and lead to a greater sense of belonging in STEM. Yet, the use of modern neurophysiological recording methodologies followed by rigorous analysis using computational tools remains relatively uncommon within undergraduate neuroscience curricula. In this article, we describe our recent development and implementation of a novel semester-long undergraduate course (*Advanced Laboratory in Neurobiology,* Haverford College) in which students experimentally compare the neurophysiological mechanisms of escape behavior of crickets and zebrafish through pharmacological intervention, extracellular recordings, and in vivo calcium imaging. Students gained mastery in hands-on scientific skills for implementing the two recording approaches and also generated their own custom-written MATLAB scripts for data analysis of both the electrophysiological and calcium imaging raw data. To foster other scientific skills throughout the course, students maintained a digital laboratory notebook, wrote and submitted a project proposal resembling a fellowship grant application, participated in a journal club discussion, and communicated their scientific findings in a final oral presentation. End-of-semester survey data indicate that our course resulted in significant growth in conceptual understanding of neurophysiological principles and skill development using scientific approaches including rigorous analysis using computational tools. Overall, this comparative neurobiology CURE represents a strong and versatile platform on which students can engage neurophysiological and computational approaches as a component of their neuroscience education.

Course-based undergraduate research experiences (CUREs) offer students the opportunity to collaboratively engage the process of scientific investigation in a hands-on manner as a means of augmenting learning (Auchincloss et al., 2014). The evidence supporting the value of CUREs across various scientific disciplines is strong: They have been shown to improve scientific literacy, critical reasoning, and course performance, and further have significant equity implications for student confidence and sense of belonging in science (Bangera & Brownell, 2014; Brownell et al., 2015; Fendos et al., 2022; Johnson et al., 2022; Olimpo et al., 2018; Thiry et al., 2012). For these reasons, there has been increasing effort to develop and implement high-quality CURE courses across the community of neuroscience educators.

Numerous neuroscience CUREs have been described focusing on a variety of techniques or model systems including the *C. elegans*, crayfish, *Drosophila*, and rodents (Cortes & Swanson, 2023; Delventhal & Steinhauer, 2020; Hall & Harrington, 2003; Kallarackal, 2023; Nesbit et al., 2015; Waddell et al., 2021). However, relatively rare among existing neurobiology CUREs is taking a comparative physiological approach in which analogous neural systems are examined with different methods and/or in different model systems. We set out to design and implement a cross-species comparative neurophysiology semester-long CURE at Haverford College in which students examined the neurophysiological mechanisms of escape behavior using both extracellular recordings of action potentials in live crickets, and *in vivo* calcium imaging in zebrafish larvae, in the context of various pharmacological interventions. While extracellular electrophysiology offers highly detailed temporal resolution by directly recording the sub-millisecond electrical changes of individual action potentials, it is often limited in spatial scale and cell-type specificity. In contrast, calcium-imaging provides a powerful, though indirect, readout of neuronal activity that potentially allows for the monitoring of larger populations of identified neurons, albeit at a lower temporal resolution due to calcium indicator kinetics. Alongside student experimentation, our course spent significant time and effort in learning computational tools to rigorously and flexibly analyze the raw physiology data from both types of experiments using MATLAB. We also aimed to determine how the guided research in this course influences student learning and growth in skills as well as sense of belonging in the neuroscience/scientific community.

In our course we examined the physiological function of neurons involved in escape behaviors in *Acheta* *domesticus*, the common house cricket, and *Danio rerio*, zebrafish larvae. Both of these models have been utilized as model systems for teaching undergraduate students (Bhatt & Challa, 2018; Dagda et al., 2013; D’Costa & Shepherd, 2009). Crickets are a widely available, cost-effective invertebrate model system in which neural activity can be examined in response to various stimuli including pharmacological intervention. Zebrafish are an accessible vertebrate model system for neuroscience research, education, and outreach (Arévalo Cuaical et al., 2024; Felzien, 2016; Forecki et al., 2023; Prakash & Toro, 2019; Shuda et al., 2024; Sorrentino, 2025). Due to their transparent early development alongside a well-developed genetic toolkit, zebrafish are an excellent system for *in vivo* microscopy including noninvasive imaging of neuronal activity via fluorescent genetically-encoded calcium indicators such as GCaMP (Doszyn et al., 2024; Vanwalleghem et al., 2018).

This CURE focused on the primary neurons involved in driving stimulus-evoked escape behavior in both systems that have evolutionary, functional, and teleological similarities. In crickets, we examined the giant interneurons within the cercal ganglion, which are involved in detecting local air pressure changes (i.e. due to a predator) sensed by the mechanosensory hairs cells on the antenna-like cerci (*Figure 1A*). When activated, the giant interneurons (red & blue, *Figure 1A*) generate action potentials to rapidly transmit that signal to the central nervous system in the head of the cricket, where neurons in the anterior ganglion activate efferent motor neurons that target muscles in the jumping leg to initiate an escape response (Kanou, 1996; Yono and Shimozawa, 2008; Insausti et al., 2011; Yamao et al., 2022). In response to local changes in air pressure, the giant interneuron action potentials can be recorded as spikes in extracellular recordings (Belosky & Delcomyn, 1977; Counter, 1976; Dagda et al., 2013) (*Figure 1A*). In zebrafish, escape-related behavior is driven by a pair of large reticulospinal neurons called Mauthner cells (red & blue, *Figure 1B*) located in the hindbrain (Jonathan & Eaton, 1989; Shimazaki et al., 2019; Takahashi et al., 2002). One of the primary sensory inputs onto the Mauthner cells comes from afferent neurons in the auditory nerve (orange, *Figure 1B*), which transmit acoustic information from mechanosensory hair cells in the otic vesicles to synapses on the large Mauthner lateral dendrites (*Figure 1B*) (Curti & Pereda, 2010). When activated by sound stimuli, a Mauthner cell sends an action potential propagating down the spinal cord to directly activate the motor neurons that innervate contralateral trunk and tail muscles to initiate a fast bend and escape swim directed away from the stimulus (Eaton et al., 1991; Liu & Hale, 2017). These ethologically relevant escape maneuvers for both species thus feature analogous giant interneurons at their core, providing a rich foundation for comparative studies.

**Figure 1.**
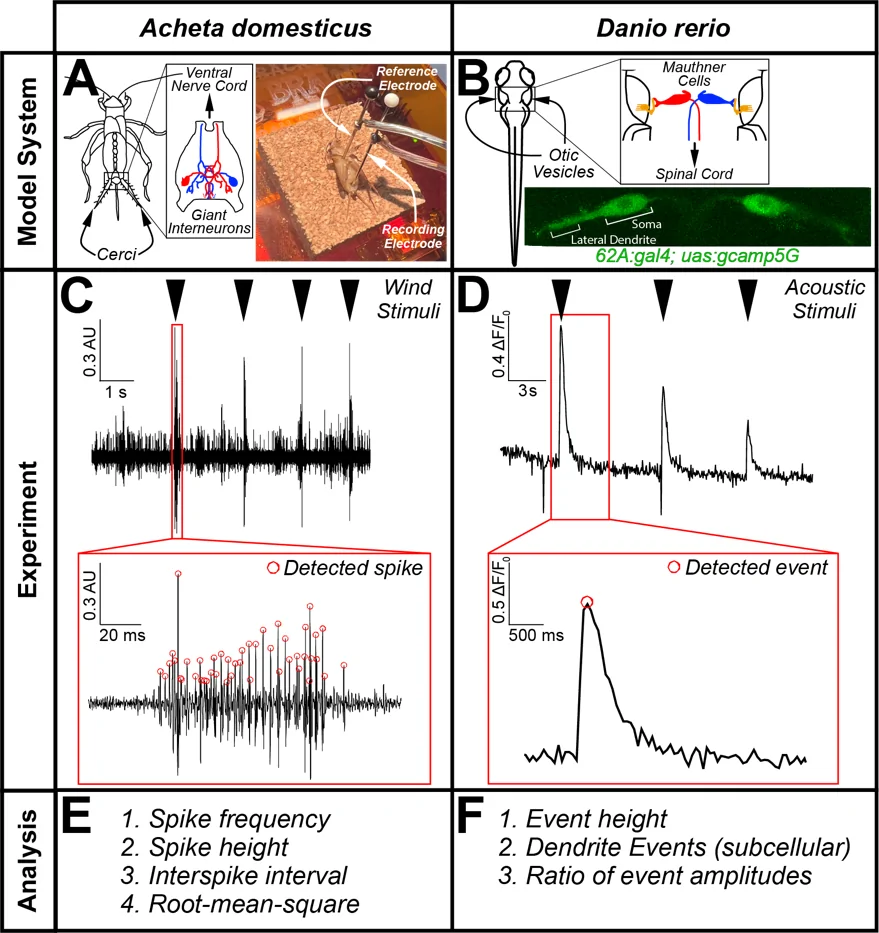
Comparative neurophysiological investigation of escape response-related neurons in *Acheta domesticus* and *Danio rerio*. *A-B.* Comparative physiology experiments were designed to investigate cricket giant interneurons *(A)* and Mauthner cells *(B)* in response to stimulation of the escape response circuitry. Diagram of representative bilateral Giant Interneurons of the cricket cercal ganglia (red and blue), along with a student-generated image of cricket recording setup is displayed in *(A)*. Diagram of Mauthner cells (red and blue) in the hindbrain receiving input at their lateral dendrites from sensory hair cells (orange) in the otic vesicles via the auditory nerve (statoacoustic ganglion, orange) is displayed in *(B)*. Representative temporal projection of Mauthner cells expressing the fluorescent calcium sensor GCaMP5G from a confocal recording of a student in the course is included in *(B)*. *C.* Representative raw data trace depicting extracellular action potentials generated in the cercal ganglion in response to wind stimulus of the cricket cercus (arrowheads). The red box displays extracellularly-recorded action potentials generated in response to a single bout of wind stimulus presentation. The voltage units are arbitrary as the .wav file condenses extracellular field potentials into -1 to 1 V range. *D.* Representative example for *in vivo* calcium imaging of Mauthner cell soma activity in zebrafish larvae. Large changes in calcium fluorescence in the soma indicative of Mauthner cell activation are observed in response to acoustic stimulation (arrowheads). The observed run-down in event magnitude is likely a combination of habituation and photobleaching of the calcium-indicator upon repeated light exposure (Marsden & Granato, 2015). Stimulus-evoked spikes and calcium fluorescence events were detected offline using custom-written MATLAB scripts by students. *E-F.* After spike/event detection various measures were used to infer biological meaning of physiological data in crickets *(E)* and zebrafish *(F)*. Example traces in *C-D* were collected by students of the course.

One of the primary barriers to conducting authentic neurophysiology research in the undergraduate classroom is the cost and expertise needed to operate the equipment involved in the electrophysiology recording and/or *in vivo* imaging set-up. In response to these challenges, many educators have pursued neurophysiology education through online simulation platforms (Crisp, 2012; Grisham et al., 2009; Heitler, 2022; Ho et al., 2021; Stuart, 2009). While these *in silico* approaches are undoubtedly valuable, especially in remote-learning conditions, we believe that experimental recording/assessing the activity in living neurons is uniquely valuable for student engagement and conceptual/technical understanding. To overcome barriers due to expensive physiology equipment, cost-effective and user-friendly electrophysiology recording hardware and software have been recently made available. Throughout the course we utilized the SpikerBox Pro hardware apparatus and the SpikeRecorder software (Backyard Brains) to acquire, digitize, and visualize extracellular recordings of cricket neurons in the cercal ganglion. In the zebrafish larvae, we used *in vivo* calcium imaging of mounted larvae (5-7 dpf). Although not ubiquitous, many primarily undergraduate institutions have access to confocal or epifluorescence microscopes and vivarium facilities for housing zebrafish to support the experiments described in this CURE.

The guiding scientific goal of our semester-long CURE was to compare and contrast the neurophysiological mechanisms involved in escape responses in crickets and zebrafish and to examine the impact of neuropharmacological agents in both systems (*Table 1*). The precise neurotransmitter systems and corresponding receptors involved in Mauthner cell and giant interneuron physiology and modulation remain unclear. Thus, students were able to conduct real-world experimentation and potentially collect empirical results that were genuinely informative in clarifying mechanisms of escape behavior across species. Overall, these approaches incorporating comparative neurophysiology experiments into a neurobiology class could be readily implemented at other predominantly undergraduate institutions and research institutes alike.

**Table 1.**
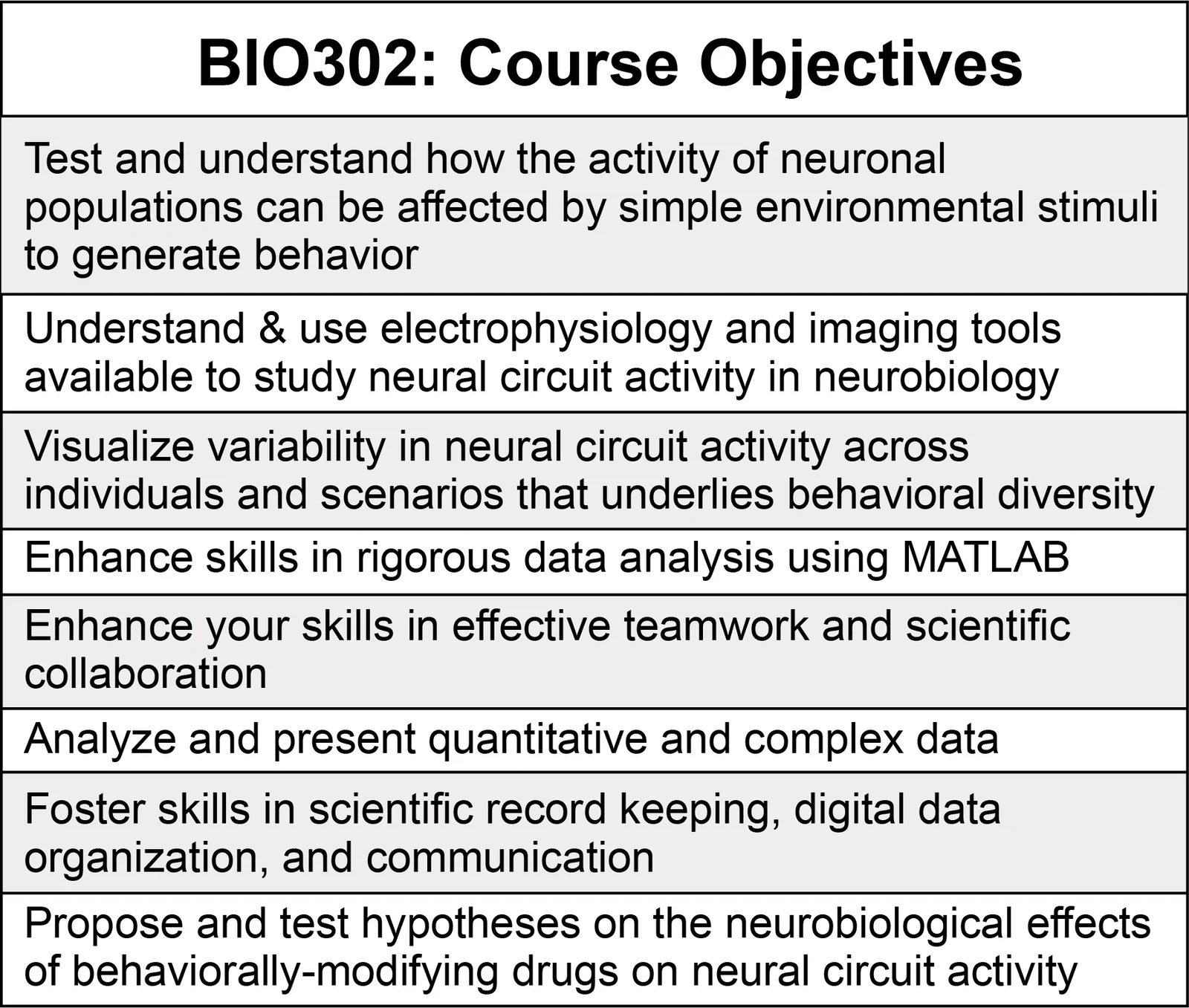
Course Objectives for BIO 302: Advanced Laboratory in Neurobiology.

## MATERIALS AND METHODS

### Course context

Haverford College is a selective coeducational liberal arts college located in suburban southeastern Pennsylvania. BIO302 is an in-person semester-long laboratory course designed for 3^rd^ and 4^th^-year students to gain experience in technical approaches utilized in neurobiology research. Each week, the course met Monday evening from 7:30-9PM to review, plan, or discuss aspects of the course that did not involve hands-on experimentation, and on Wednesdays and Fridays from 1-4PM for laboratory experimentation and other activities. The course had two instructors: an Associate Professor and Chair of Biology (RAJ), and a Visiting Assistant Professor of Biology (ERW), as well as two 4^th^-year students as teaching assistants (TAs). There were ten students enrolled in the course, and all students completed the entire semester with a passing grade.

### Data collection and analysis

The student survey components of our study were reviewed by the Haverford College Institutional Review Board and declared exempt under category 1: “research, conducted in established or commonly accepted educational settings, that specifically involves normal educational practices that are not likely to adversely impact students’ opportunity to learn required educational content or the assessment of educators who provide instruction.” At the end of the semester and after all work had been submitted, students were prompted to complete a voluntary anonymous survey unconnected to their course grade, administered by Haverford College support staff. Eight of ten students completed the survey. Students were asked to provide general feedback on the course, their proficiency in various scientific skills before and after the course, as well as their sense of identity as a scientist and belonging in Science, Technology, Engineering, and Mathematics (STEM). We analyzed and visualized aggregate student response data using Prism 10.6 (GraphPad).

### Course objectives, activities, and assessments

To reflect an authentic scientific research environment, our course objectives were designed without separation into distinct content and process categories. Instead, learning goals encompassed aspects of neurobiological content as well as technical mastery (*Table 1*).

The research aims of our CURE were to comparatively examine the neurophysiological basis of neurons involved in escape behavior in crickets using electrophysiological recordings and in zebrafish larvae via calcium imaging using confocal microscopy. To analyze these data in a rigorous, reproducible, and flexible manner, students were guided in creating their own custom-written MATLAB scripts for the analysis of raw data. The course, therefore, was naturally divided into two phases (*Table 2*). The first phase (weeks 1-6) focused primarily on technical training, exploration of the techniques involved in data collection and analysis in both model systems, and building a background understanding of neurotransmitter systems and the behavioral context. In the second phase (weeks 7-14) students were divided into three groups to design, implement, analyze, and present an original hypothesis-driven research project utilizing all the tools gained in Phase 1.

**Table 2.**
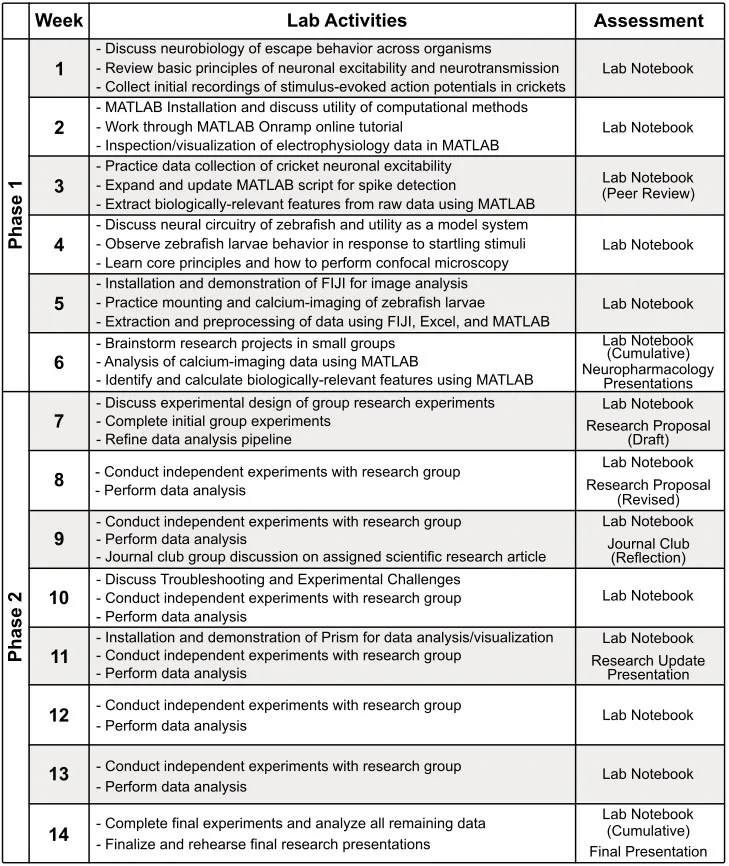
Timeline of weekly lab activities and student assessments.

A key goal of the course was to model and foster best practices for designing and recording rigorous and reproducible science. Thus, each student was assigned an electronic laboratory notebook at the beginning of the class in the form of a shared Google Drive document, designed by the instructors with simple templates and prompts for daily entries to fully document their work (*Supplementary File 1*). Students were responsible for updating their lab notebook for each class session to include Purpose, Protocol, Results, and Discussion sections. Template tables for cricket physiology and zebrafish imaging experiments were provided in the lab notebook to help guide students in identifying and recording the critical variables to track in these techniques, and to foster a standardized manner of data nomenclature and collection across the whole course. To maximize transparency and accessibility of data, each student would upload all recorded data files into a centralized course Google Drive folder, hyperlink the data within their lab notebook, and hyperlink to relevant entries or information in the notebooks of classmates in their group. The online digital nature of these lab notebooks facilitated regular weekly feedback by student TAs via comments flagging items where additional detail should be included, instances of missing or unlinked digital data, as well as components that were effective or well-considered, earning weekly completion-based course credit (see *Supplementary File 2*). Students also gave and received guided constructive peer feedback after three weeks to allow them to learn from the approaches of other students, and received more aggregate larger-scale feedback on their notebooks through ungraded 10-minute “check-in” discussions with the faculty twice during the semester, and through graded reviews from the course faculty midsemester and at the end of the course.

One of the course learning goals was to generate and experimentally test hypotheses regarding pharmacological agents on analogous neural circuits within the cricket and zebrafish systems (*Table 1*). The student groups were responsible for selecting a particular pharmacological agent based on their exploration of the scientific literature and their interests. In the second phase of the course, students were prompted to choose a pharmacological agent from a curated list of IACUC-approved chemicals. Working in their final groups, students investigated known mechanisms of these agents through literature searches, and developed clear and testable hypotheses to direct novel experiments that they assembled into a written proposal (see *Supplementary File 3* for structure). Groups received feedback on a proposal draft, with guidance on clarifying specifics of their resource needs and timing, then they updated their proposal and began their group experimental plan. A full list of reagents and equipment used for experimentation is provided, including the approved list of possible pharmacological agents (*Supplementary File 4*).

Class assessments took a variety of forms and were designed to foster the development of scientific skills including collaborative work, engagement with scientific primary literature, communication of scientific findings, articulation of experimental design and hypotheses, and keeping a detailed laboratory notebook. To reflect these goals, weekly completion grading and regular narrative feedback was provided on entries from each laboratory session in electronic lab notebooks (see example entries in *Supplementary File 2*). In week three, students were tasked with providing peer review feedback on their classmates’ lab notebooks to better engage the peer review process and facilitate the sharing of best-practices in lab notebook maintenance and design. In week six, each student gave a short oral presentation on one of the IACUC-approved neuropharmacological agents to help inform their selection of agents for testing in cricket and zebrafish escape behavior. After being divided into three small groups for the remainder of the course, students were tasked with composing a research proposal in the style of a grant application in which they stated a chosen overarching question involving the impact of a neuropharmacological agent on the neural mechanisms of escape behavior and proposed a detailed experimental protocol to rigorously test their hypothesis (*Supplementary File 3*). Students engaged with primary scientific literature throughout the course, and led a journal club in week nine on an assigned research article relevant to the system and modeling potential data presentation approaches (Marsden & Granato, 2015). Students were instructed to guide their classmates through the figures and offer well-reasoned scientific criticisms of the work. The final assessments of the class were the research project update and final presentation in which the groups showcased their own experimental findings with prepared graphic visualizations and statistical comparisons. The final presentation took place on the final day of class and as an overall culminating experience served as the final examination. Together, these assessments incentivized students to grow in a diverse range of useful scientific skills. Please contact the corresponding author for copies of any student assessments, rubrics, or surveys used in this course.

### Animal care and handling

Live medium-to-large crickets were obtained the morning of each class session from a local pet store, and maintained in an inflated plastic bag. Individuals were retrieved for experimental use from the bag using forceps to grab an individual by the jumping leg, and were then immediately anesthetized by submerging in ice water (see details below).

Zebrafish (*Danio rerio*) transgenic lines were maintained in the Tüpfel long fin (TLF) background, and raised on a 14 h:10 h light:dark cycle as previously described (Jain et al., 2018). Fertilized embryos were raised in 1x E3 embryo medium (5 mM NaCl, 0.17 mM KCl, 0.33 mM CaCl_2_, 0.33 mM MgSO_4_) and maintained at 29ºC on the same light cycle through imaging (5-7 dpf). Embryo media was transiently supplemented with 10-15µg/ml pronase (Sigma) at 1 day post fertilization (dpf) for 12-24 hours to weaken chorions and support synchronized hatching. Starting at 24 hpf fish were kept in E3 media supplemented with 200µM phenyl thiourea (PTU, Sigma) to prevent pigmented melanophore development and enhance transparency for live imaging. At 2 dpf, healthy fish were separated into 10cm petri dishes of no more than 75 individuals per dish in fresh 1x PTU/E3 media. Sex is not determined in zebrafish until 25-60 dpf so analyses of larvae were performed on all individuals without consideration of sex. For course implementation, crossing and husbandry prior to 5 dpf was performed by the course faculty and TAs, and enrolled students began handling and maintaining larvae at 5 dpf. Following experiments at 5-7 dpf, fish larvae were humanely euthanized at the conclusion of the experimental day by the rapid chilling method. All procedures using zebrafish were approved by the Haverford College Institutional Animal Care and Use Committee.

### Electrophysiological recordings of neuronal spiking behavior in the cricket cercal ganglion

Extracellular recordings of neural excitability in the cercal ganglion of the cricket were completed by adapting previously published protocols (Dagda et al., 2013). Briefly, students anesthetized crickets by submerging them in ice water for ~90 seconds. They then mounted the cricket to a piece of corkboard with two electrode pins—one placed through the coxa and one placed through the cercal ganglion in the posterior end of the cricket abdomen *(Figure 1A)*. Electrical signals were amplified, filtered (low/high-pass filtered at 160/3700 Hz), and digitized (Neuron Spikerbox Pro, Backyard Brains) and visualized in real-time using SpikeRecorder software (Marzullo & Gage, 2012). Because neurons in the cercal ganglion respond to pressure changes to detect predators and initiate a jumping response, extracellular spikes were elicited by consistent stimuli delivered through pipet-induced wind stimulation or via speakers placed a few cm away from the cricket as done previously (Belosky & Delcomyn, 1977; Counter, 1976). The effect of neuropharmacological agents was determined by recording the cricket before and after abdominal injection of the agent (MK-801, a potent noncompetitive antagonist of NMDA-type glutamate receptors, or fluoxetine, a selective serotonin reuptake inhibitor so as to augment serotonergic signaling) or the related vehicle control (water, 1% DMSO, etc.) which usually occurred a few minutes after the baseline untreated cricket recording. Student groups selected their neuropharmacological agent from the list of approved drugs (*Supplementary File 4*). Anesthetized crickets were euthanized at the conclusion of the experiment by rapid decapitation with a sharp razor blade.

### *In vivo* calcium imaging of zebrafish Mauthner cells

*In vivo* calcium imaging of Mauthner cells in zebrafish larvae was performed as previously described (Marsden & Granato, 2015). Zebrafish larvae carrying the transgenes *nkhspGFF62ATg* [ZFIN identifier: ZDB-ALT-150717-1] and *Tg(UAS:GCaMP5G)a151* [ZFIN identifier: ZDB-ALT-151028-4], abbreviated as *62A:gal4; uas:gcamp5G,* were used to drive the genetically-encoded calcium indicator GCaMP5G specifically in Mauthner cells (Lacoste et al., 2015; Yamanaka et al., 2013). Fish carrying the transgene *TgBAC(slc6a5:Gal4FF)nns22* [ZFIN identifier: ZDB-ALT-131127-3], also referred to as *glyT2:gal4,* were also used by a student group during piloting, to drive expression of GCaMP5G in glycinergic inhibitory interneurons (Satou et al., 2013). Zebrafish transgenic lines may be requested from their source laboratories, and/or from the Zebrafish International Resource Center (ZIRC, https://zebrafish.org). Experimental unanesthetized zebrafish larvae raised in 1x PTU/E3 (5-7 dpf) were transferred to a microfuge tube of 2% low-melt agarose at 42ºC in a minimal volume of media using a p200 micropipet with a cut tip to produce a wider bore. Larvae were oriented dorsal-side-down in coverslip-bottom imaging dishes (MatTek) by students, using extra thin gel-loading tips to manipulate them into the desired orientation under a simple transmitted light dissecting microscope (Leica GZ6), then overlayed in the dish with 1xPTU/E3. Time-lapse image sequences were acquired using a Leica Stellaris 5 inverted line-scanning confocal microscope (18-20 frames per second). Students piloted diverse approaches to providing acoustic stimuli, including Python-generated waveforms and free sound files found online, through small speakers such as USB speakers (Lielongren) or a simple speaker wired through an amplifier SC 5.9 OM - 8 Ohm (Visaton), directed at the stage and/or imaging dish. Assessments of the effects of the same neuropharmacological agents used in crickets (MK-801 and fluoxetine) were completed by collecting recordings before and after treatment (typically 20-30 minutes, though a consistent incubation time was used within each group) with neuropharmacological agents or the associated vehicle control directly added to the bath solution surrounding the mounted fish. As with the cricket experiments, student groups selected their neuropharmacological agent from the list of approved drugs (*Supplementary File 4*).

### Data processing/analysis

Through the course, students gained basic competence in using MATLAB (2023a, MathWorks) to rigorously and flexibly extract biological meaning from raw data recordings in both experimental systems. Learning MATLAB syntax and programming strategy for data analysis was facilitated by students completing the MATLAB Onramp online tutorial (https://matlabacademy.mathworks.com/details/matlab-onramp/gettingstarted), and supplemented with numerous guided in-class exercises using their own experimental raw data. Students independently composed two MATLAB analysis scripts, one for each model system and data type, and collaboratively used them throughout their entire research project to ensure rigorous analysis and reproducibility. Example code is included in the student notebook entry in *Supplementary File 2*.

The SpikeRecorder acquisition software records normalized (-1 to 1) voltage information as a .wav audio file which was imported into MATLAB using the *audioread* function. Identification of extracellularly-recorded action potentials was accomplished using the *findpeaks* function and various properties were calculated including frequency, interspike interval variability, width of the AP waveform, root-mean-square, etc. Plotting of traces with detected peaks allowed for manual inspection of traces to ensure proper detection of spikes. After using MATLAB to extract these biological informative measures, the post-processing data were analyzed in GraphPad Prism to compare across before and after treatment with vehicle and some other neuropharmacological agent.

MATLAB-based analysis of the *in vivo* calcium imaging data was conducted similarly. Students first utilized FIJI/Image J (Schindelin et al., 2012) to generate a maximum intensity temporal projection of the recording on which to draw regions of interest (ROIs) around fluorescent Mauthner cells and an unlabeled brain region as a background control, then extracted the average intensity values of the ROIs over the entire time series (usually ~1 minute). These average intensity values were then exported from FIJI as a .csv file and imported into MATLAB using the *readmatrix* function or the built-in import tool. Students designed their scripts to correct the raw intensity values for neurons with the background and transform them into a plot of ∆F/F_o_ over the time series in which students were able to identify peak values using the *findpeaks* function. Similar to their cricket physiology experiments, students extracted biologically informative values based on their peak detection such as stimulus-evoked peak height, decay time constant, subcellular dendritic calcium transients, etc., and then analyzed offline in GraphPad Prism to compare the values before and after treatment with vehicle or neuropharmacological agent. *Figure 1* displays the overall experimental pipeline from raw data collection to final output measures for plotting and analysis in GraphPad Prism for both experimental model systems.

## RESULTS

Each of the three student groups made substantial progress in examining the impact of a neuropharmacological agent on the neurophysiological responses of giant interneurons and Mauthner cells when exposed to escape-inducing startling stimuli. Two groups investigated MK-801, an NMDA-type glutamate receptor antagonist, and did not detect significant alterations in the neuronal excitability contributing to escape-related neuronal activity in either crickets or zebrafish, suggesting that NMDA-type receptors may be dispensable for sound/air pressure-evoked activation of either giant interneurons or Mauthner cells. Similarly, one group’s experiments with direct application of glutamate produced no effect in crickets, while zebrafish results were unclear due to low sample size. While this contrasts with prior behavioral reports of increased Mauthner-dependent startle response initiation after MK-801 treatment (Wolman et al., 2011), this may reflect the different type or intensity of stimuli used here (Deslauriers et al., 2024) or low sample sizes producing low statistical power in some analyses (*Supplementary File 5*). Also, students did not detect significant MK-801 induced changes to ΔF/F_0_ in the Mauthner soma or lateral dendrite in response to individual acoustic stimuli which is consistent with previous calcium imaging data (Marsden & Granato, 2015). Among the students examining MK-801, there was a desire for future experiments to interrogate other glutamate receptors to better appreciate the full role of glutamate in the escape behavior. Example student-generated results and analyses excerpted from final presentations are shared in *Supplementary File 5*. Further, one group investigated the impact of fluoxetine, a selective serotonin reuptake inhibitor, and also did not identify any significant alterations in giant interneuron function by electrophysiology or in Mauthner cell activity by *in vivo* calcium imaging (*Supplementary File 5, Panel F*). Overall, these results suggest that if serotonergic modulation impacts escape behavior in crickets or zebrafish larvae, it acts through mechanisms other than direct effects on excitability of giant interneurons and Mauthner cells, or at a different timescale than explored in these experiments. Together, these results, albeit negative findings, provide authentic insight into the neurophysiology of escape behavior in both crickets and zebrafish, and offered opportunities for students to evaluate their work in the context of published literature.

The course digital lab notebooks facilitated multiple aspects of student learning throughout the course. Weekly TA comments and completion grades supported regular as-you-go data recording and regular reflection on the experimental process. Students were encouraged to add comments tagging faculty and TAs where they had questions about information, details, or expectations. Such in-notebook comments automatically emailed faculty and TAs with the questions, allowing rapid and targeted feedback in real-time. This interactive approach encouraged students to clearly articulate questions arising during analyses and any out-of-class work, and helped to surface their individual learning arcs. Example student-generated notebook entries with TA feedback are presented in *Supplementary File 2* (see 2023-11-08 entry).

**Figure 2.**
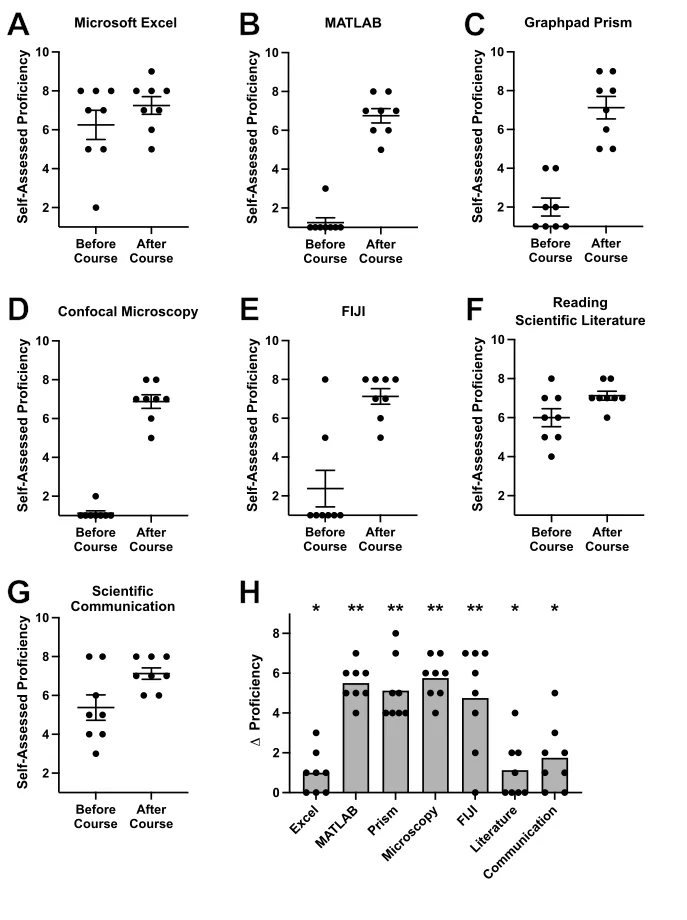
Post-course survey results indicate growth in scientific skills. *A-G.* Students were asked to rate their proficiency in a specific scientific skill or technique *Before* and *After* completing the CURE with a score of 1 being *not proficient at all* and a score of 10 being *extremely proficient* for: Microsoft Excel *(A)*, MathWorks MATLAB *(B)*, GraphPad Prism *(C)*, confocal microscopy *(D)*, FIJI *(E)*, reading scientific literature *(F)*, and scientific communication *(G)*. Line represents average score ± SEM of the 8 responses. *H.* Change in self-reported proficiency after the course relative to before the course. Bars depict class average values and each point represents an individual student. *p<0.05, **p<0.01 for corrected Wilcoxon matched pair signed rank test.

After the course, students took a survey and rated their proficiency in various important scientific skills and techniques on a scale from 1 (*not* *proficient at all*) to 10 (*extremely* *proficient*) reflecting on their ability levels before and after the course (*Figure 2*). Self-assessed gains in proficiency were observed for each scientific skill measured in the survey (*Figure 2A-G*). We assessed these proficiency gains via multiple Wilcoxon matched-pairs signed rank tests, and the presented p-values were corrected by the two-stage step-up (Benjamini et al., 2006) method using a False Discovery Rate Q = 5%. In alignment with the primary technical objectives of the course, students reported the greatest gains in MATLAB and confocal microscopy (*Figure 2H*). Growth in skills focusing on data analysis software (FIJI and GraphPad Prism) were also strongly improved while more modest gains were seen for the use of Microsoft Excel and the reading of scientific literature and communication of scientific results. These survey results indicate that the primary student learning goals were achieved throughout the CURE.

**Figure 3.**
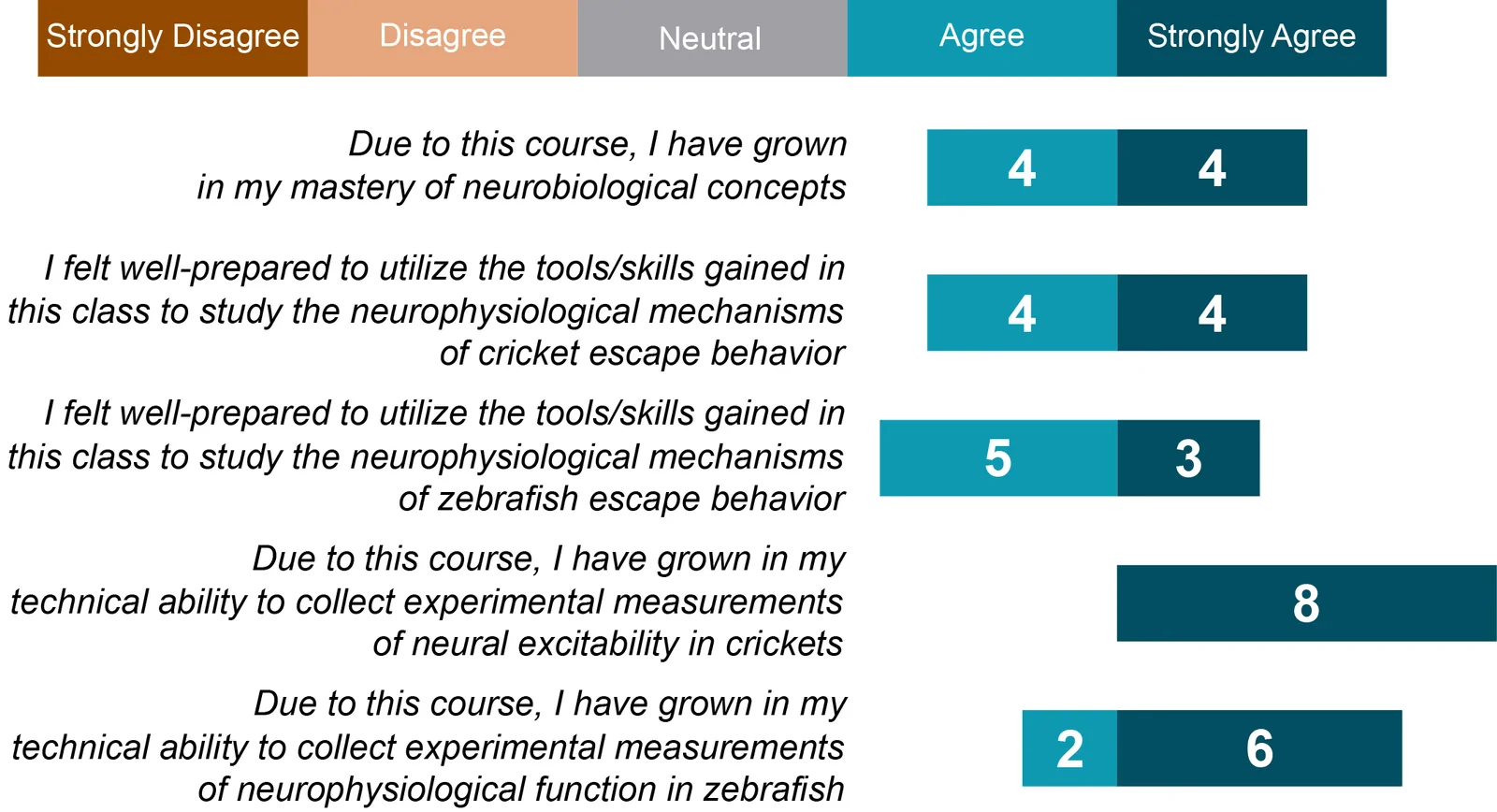
Post-course student survey reveals growth in conceptual understanding and experimental ability in neurophysiology of crickets and zebrafish.

Students reported substantial confidence and enhancement of their technical and conceptual understanding of neurophysiological concepts and techniques in the invertebrate and vertebrate model systems used. In the post-course survey, students responded to statements about these concepts and techniques using a modified Likert scale (Strongly Disagree, Disagree, Neutral, Agree, Strongly Agree; *Figure 3*). At the start of the course, all students indicated they had no prior direct experience with electrophysiology or calcium imaging; thus these responses indicate clear advances in these technical learning goals.

**Figure 4.**
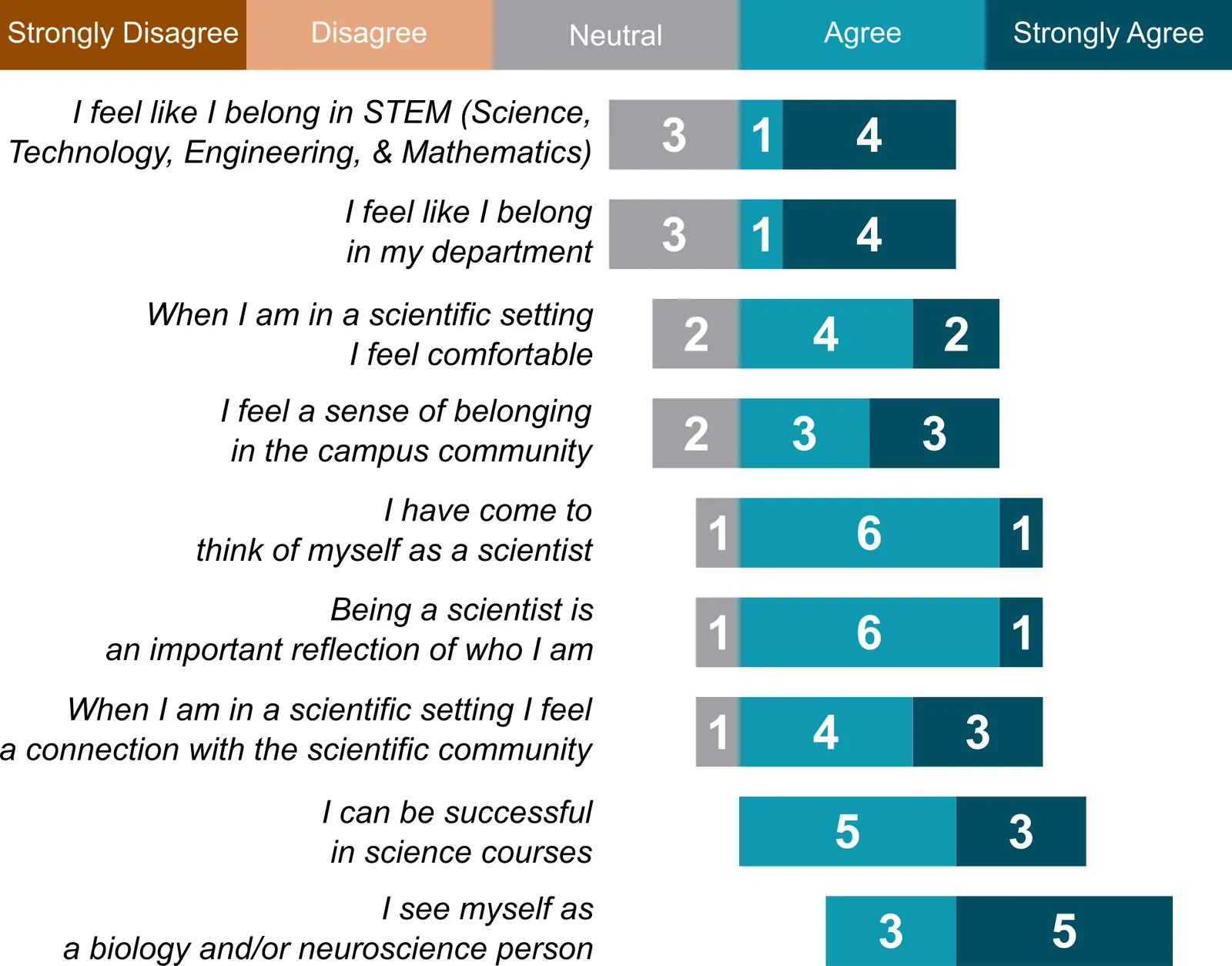
Post-course student survey reveals a strong sense of biology/neuroscience identity.

In the post-course survey, students also responded to statements about their sense of scientific identity using a modified Likert scale (Strongly Disagree, Disagree, Neutral, Agree, Strongly Agree). In general, students showed a strong sense of scientific identity considering that the majority of responses to each statement was affirmative. Interestingly, our results showed the most affirmative responses to the statements, *“I can be successful in science courses”* and *“I see myself as a biology and/or neuroscience person”* (*Figure 4*). While we are unable to draw conclusions related to the causality of participation in this CURE and an increased sense of belonging in STEM, our data are consistent with an improved sense of identity as a biologist/neuroscientist, considering that these statements were more affirmed than the more general alternatives *“I feel like I belong in STEM”* or *“I feel like I belong in my department”* (*Figure 4*).

Student narrative responses provide potential insight into how the course delivered on its goals for scientific growth and support of STEM identity. In response to the prompt “*What aspects of the course do you believe worked well, and why?”,* many students identified the overall structure and the sense of autonomy and creativity in facilitating a strong learning environment conducive to growth. For example, one representative response articulated:”*I think the structure of the class was great. One thing I hadn’t gotten in other classes was that I was able to comprehend and digest the goals of the class as well as week to week.”* Another response stated, *“I believe that the relative autonomy was a good model for working through troubleshooting and finding creative solutions to problems.”* Overall, these results indicate that the course delivered on its objectives to foster student engagement in an authentic research experience marked by autonomous experimentation, collaboration, and hands-on scientific learning.

## DISCUSSION

In this report, we describe our efforts to design, implement, and assess a novel and reproducible CURE focusing on the neurophysiological mechanisms of escape behavior. Relative to existing courses, we believe that our course is uniquely well-suited for student engagement and learning in numerous ways. For one, our course was designed to be comparative in nature: We utilized two distinct model systems, one invertebrate (adult crickets), and one vertebrate (zebrafish larvae). Our comparative ethos was mirrored in our use of two different methods to rigorously record and quantify neuronal activity/excitability via extracellular electrophysiology and *in vivo* calcium imaging. Students were regularly encouraged to forge connections across species and approaches and identify general principles of escape behavior in animals (i.e. optimized for speed, engaging relatively few total neurons, directly connected to escape-related muscles, etc.) and further consider important technical considerations for the varying ways of looking at neurophysiological function in crickets and zebrafish (temporal resolution of recording method, difficulty dealing with signal noise, etc.). Logistically, engaging two distinct experimental systems helped allow groups to stagger use of bottleneck equipment (i.e. the confocal microscope). Pedagogically, comparative neurobiology frameworks such as the one described here can foster a well-connected understanding of overall biological principles, rather than rote mastery of a single isolated technique in a single model system.

### Building computational skills

A throughline of our entire CURE was developing and implementing experimental data analysis skills via programming in MATLAB. Computational and data science skills and approaches, such as programming in MATLAB, are growing in their relevance and ubiquity in the scientific community, yet undergraduate neuroscience courses do not yet consistently incorporate these skills into the curricula (Goldman & Fee, 2017; Grisham et al., 2021; Hoy, 2021; Juavinett, 2022; McDonald et al., 2022). Our course provided an excellent platform for students to learn basic programming in MATLAB through the design and implementation of their own data analysis. Within 14 weeks, each student was able to gain basic proficiency in using diverse MATLAB functions, generate their own scripts to visualize their raw data, and practice designing code to solve authentic problems in their own data analysis (see example student-generated code and adaptations in *Supplementary File 2*, 2023-11-03 entry) as opposed to working with pre-curated data sets, pipelines, or problems only in the abstract. Although learning MATLAB was inherently challenging for students without any prior coding experience (in our case the majority of the class, *Figure 2B*), allowing multiple weeks for students in groups to learn the basic skills interspersed with data collection helped to manage frustration (*Table 2*), and students were uniquely motivated to gain these skills when they could see the direct link between their own experimentation and how MATLAB allowed them to gain access to the overall biological meaning contained within the raw data. Furthermore, since students used MATLAB to comparatively analyze different but analogous experimental data (i.e. evoked extracellular field potential spikes versus intracellular fluorescence intensity changes, *Figure 1C-D*), they could build on and adapt their group’s existing code when switching to a new data type rather than starting from scratch. The analogous data cleaning and handling needs of related experimental methods also provided students an opportunity to experience firsthand how neuroscience theory interfaces with distinct yet related neural activity monitoring approaches.

### Building experimental design skills

Lastly, we incorporated authentic scientific experimentation into our course by enabling students the task of generating and testing their own novel hypotheses regarding the impact of various neuropharmacological agents on the neuronal mechanisms involved in escape behavior in crickets and zebrafish larvae. Given the open-ended nature of these questions, students were excited to creatively design and perform experiments to learn something new about these systems. Encouraging piloting in Phase 1 and experimental replication in the flexible timed Phase 2 offered opportunities for students to build confidence in the diverse techniques (*Figure 2B, D, E*), and empowered them to build realistic experimental planning and management skills. In our implementation, the scientific results of each of the three groups in this course were negative in their nature (i.e. the drugs did not lead to a consistent impact on neuronal excitability in either zebrafish larvae or crickets). For this reason, future improvements on this CURE could encourage students to identify and utilize appropriate negative and positive controls to better interpret results. For example, in cricket recordings, students could use nicotine, an agent known to excite neurons of the cercal ganglion, as a positive control, as they did during Phase 1 of our course, and use CaCl_2_ or other blockers of synaptic neurotransmission to better ensure the sensitivity of the experiment to potentially detect biologically meaningful results (Dagda et al., 2013).

As in any authentic scientific research experience, technical troubleshooting was a consistent feature of the course. Rather than shield students from troubleshooting technical challenges, we sought to walk through the troubleshooting process alongside students. Technical troubleshooting should be expected through the course including in data acquisition of electrophysiological and calcium imaging recordings, developing and optimizing stimulus delivery approaches, data file management and sharing, raw data analysis using MATLAB, and in the visualizing and statistically analyzing data using Microsoft Excel and GraphPad Prism. These moments of overcoming technical and/or conceptual challenges offered excellent collaborative learning opportunities for students to think deeply about the nature of specific problems and creatively brainstorm and attempt work-around solutions (for example, see Discussion sections of the example student notebook entries in *Supplementary File 2*).

### Variations in the course

The CURE described here is flexible and is suitable for adaptation in various ways. For one, the same experimental set-up could be utilized to investigate neuropharmacological agents other than MK-801 and fluoxetine as was the case in our course. Modulators of voltage-gated ion channels would be particularly of interest and would likely be more likely to yield significant direct impacts on cellular excitability than perhaps was seen in our course considering MK-801 and fluoxetine act as neuromodulators of glutamatergic and serotonergic neurotransmission respectively. Other adaptations could include examining the impact of different types of stimuli for eliciting escape-related behavior. Here we used presentations of wind stimuli or specific sounds to activate the crickets and zebrafish larvae. However, different properties of the stimuli (duration, intensity, frequency) could be explored by the students before selection of a single stimulus as part of the pilot phase of the experimental design.

Further, although their clearly identifiable morphology and location make the Mauthner cells particularly tractable for students, the wealth of alternative zebrafish transgenic driver lines allows flexibility to express GCaMP in other neural components of zebrafish escape circuitry, which may be requested from the generating labs and/or the Zebrafish International Resource Center (Marquart et al., 2015). While we used confocal imaging for this course iteration, this approach is flexible to carry out through epifluorescence imaging, as other students obtained similar calcium fluctuation data in Mauthner cells using the same transgenic line on a Nikon Eclipse TE-2000S inverted epifluorescence microscope with a digital camera (pco.edge 4.2 LT) and Nikon’s NIS Elements software in a different course led by author RAJ. While we used MATLAB and Prism for data analysis due to available on-site student MATLAB licenses and free teaching licenses provided by GraphPad, other free software could be used to similar effect such as Python, R Studio, and Jamovi. Lastly, we also envision that other technical approaches could be added to the class to augment the learning experience: histological approaches to visualize escape-related neurons in both systems, rigorous quantification of animal behavior using one of many free tracking software options (Burgess & Granato, 2007; Gallois & Candelier, 2021; Mathis et al., 2018; Mirat et al., 2013; Pennington et al., 2019; Pittman & Ichikawa, 2013; Truong et al., 2024; Walter & Couzin, 2021), and measurements of simple non-associative learning such as habituation and/or sensitization (Wolman et al., 2011).

### Implementation Challenges and Recommendations

Based on our experience, successful implementation of this CURE course requires proactive management of technical and logistical challenges. For example, the significant difficulty for students in learning MATLAB necessitates a curriculum that manages student frustration and is flexible for diverse learning speeds, such as through staggering days of learning coding with hands-on experimental days. As would be expected, both troubleshooting of the technical equipment and computational approaches requires instructor knowledge and equipment familiarity so that potential issues can be overcome and the original hurdle can be turned into a learning experience for the student. Sharing, accessing, and retaining extensive digital data and analyses can be a challenge to wrangle. We recommend instructors consider this in advance and establish consistent online folders for students to deposit their files as they go, hyperlinking to them in their digital lab notebooks. We explicitly included this digital data management in the lab notebook scoring rubric to make visible this critical aspect of work, and recommend instructors regularly download backups of these centralized data repositories. There are also other practical considerations for this course. For example, the instructors must plan ahead to ensure that zebrafish larvae are available at the proper ages when they are needed for experiments. In our course, having two instructors was logistically helpful so that we could avoid bottlenecks in using high-end equipment such as the confocal microscope, though experienced undergraduate teaching assistants also are effective in this regard. Overall, we recommend that instructors seeking to implement this course practice each of the techniques described so that they are well familiar with the technique-specific troubleshooting challenges and learning opportunities.

### Implications for neuroscience education

Our survey results indicated that this course was successful in spurring student growth in neuroscience techniques and concepts. The areas of greatest growth, MATLAB and confocal microscopy (*Figure 2H*), were indeed the primary focus of the course, with substantial time allocated for learning and implementing these methodologies. The more modest gains reported in literature review and scientific communication likely reflect a higher baseline proficiency in these skills among upper-level undergraduates, whereas the intensive, iterative nature of the course was specifically designed to bridge the larger gap in specialized technical and computational expertise. Nonetheless, students also reported growth in every scientific aspect probed by the survey. Beyond reported growth, our queries regarding sense of identity as a scientist were supportive of our general goal of fostering an inclusive scientific research environment. Although not directly asked by our post-course survey, narrative feedback from students also indicated a strong sense of classroom community and collaborative teamwork. While further studies are needed to compare how this course serves student learning and sense of identity relative to other traditional and/or hands-on CUREs, our results support the effectiveness of this course design for student learning.

### Conclusion

For all the above reasons, we believe that this course and adaptations thereof comprise a solid scaffold for neuroscience educators to provide students with opportunities to conduct engaging neurophysiological and rigorous computational research approaches in the laboratory setting to serve a well-rounded neurobiology education.

### ADDRESS CORRESPONDENCE TO:

Dr. Roshan Jain, Department of Biology, Haverford College, 370 Lancaster Ave., Haverford, PA 19041. Email: rjain1@haverford.edu

Dr. Eric Wengert, Department of Medical Education, Geisinger College of Health Sciences, 525 Pine St. Scranton, PA 18509. Email: erwengert@geisinger.edu

## Supplementary Material

Supplementary File 1 - Digital Notebook Template

Supplementary File 2 - Example Lab Notebook Entries

Supplementary File 3 - Research Proposal Instructions & Rubric

Supplementary File 4 - Materials List

Supplementary File 5 - Sample Student Analyses
