# Supplementary material for: Comparative Investigations of the Neurophysiological Mechanisms of Escape Behavior: A Course-based Undergraduate Research Experience
Source: J Undergrad Neurosci Educ. 2026 Jun 4;24(2):1–16. doi: 10.59390/001c.160661 (PMC13625905; doi:10.59390/001c.160661)
Supplement: Supplementary file 1 [file junejournal_2026_24_2_160661_340112.pdf]

Table Of Contents

2023-11-03: Calcium Imaging Analysis from Initial Piloting & Piloting Glutamate Treatment in Crickets 2

2023-11-08: Day 3 of Group Research Project for Collection of Cricket Data with Replicate Crickets Testing MK-801 Concentrations 10

Cricket Pharmacology/EPHYS Experiment 11/8/23 11

2023-12-01: Testing the Effect of MK-801 & Glutamate Treatment on Mauthner Neurons 15

2023-11-03: Calcium Imaging Analysis from Initial Piloting & Piloting Glutamate Treatment in Crickets

Purpose:
After our trial run with the GCaMP5G calcium imaging of the zebrafish larvae, we will be analyzing some of the recordings where neural activity was observed during exposure to the acoustic stimuli using FIJI and MATLAB. Additionally, we are moving on to piloting the Glutamate treatment in the *Acheta domesticus* crickets to assess the role of NMDA receptors' activation in startle response.

Protocol:

Calcium Fluorescence Imaging Analysis

- Calcium imaging analysis was done using the Analysis in FIJI and Analysis in MATLAB protocol used on 10_04_2023, with the following additions made:
    1. After selecting Max capacity, go to Image < Adjust < Brightness/ Contrast, and adjust the maximum brightness, in order to clearly see the contrast in the different glycinergic neurons.

Glutamate Treatment in Crickets

1. Set up the Spiker Box and Spike Recorder using the setup protocol performed on 09_08_2023.
2. Start off by testing a 1:10 and 1:100 dilution of the 25mM Glutamate stock solution for a total volume of 1mL.
    - **Note:** The 25mM Glutamate stock solution was not completely dissolved in the water, so concentrations of the dilutions might be a little off.
3. Anesthetize the cricket using the Anesthesia protocol and place the reference electrode in between the hind legs, and the recording electrode at the terminal abdominal ganglion.
4. ~3-4min after anesthesia, if the cricket is responsive, perform an electrophysiological recording on the cricket exposed to the stimulus, prior to any treatments.
5. Inject the cricket with 10-25ul of the desired treatment, and wait 2 minutes before recording.

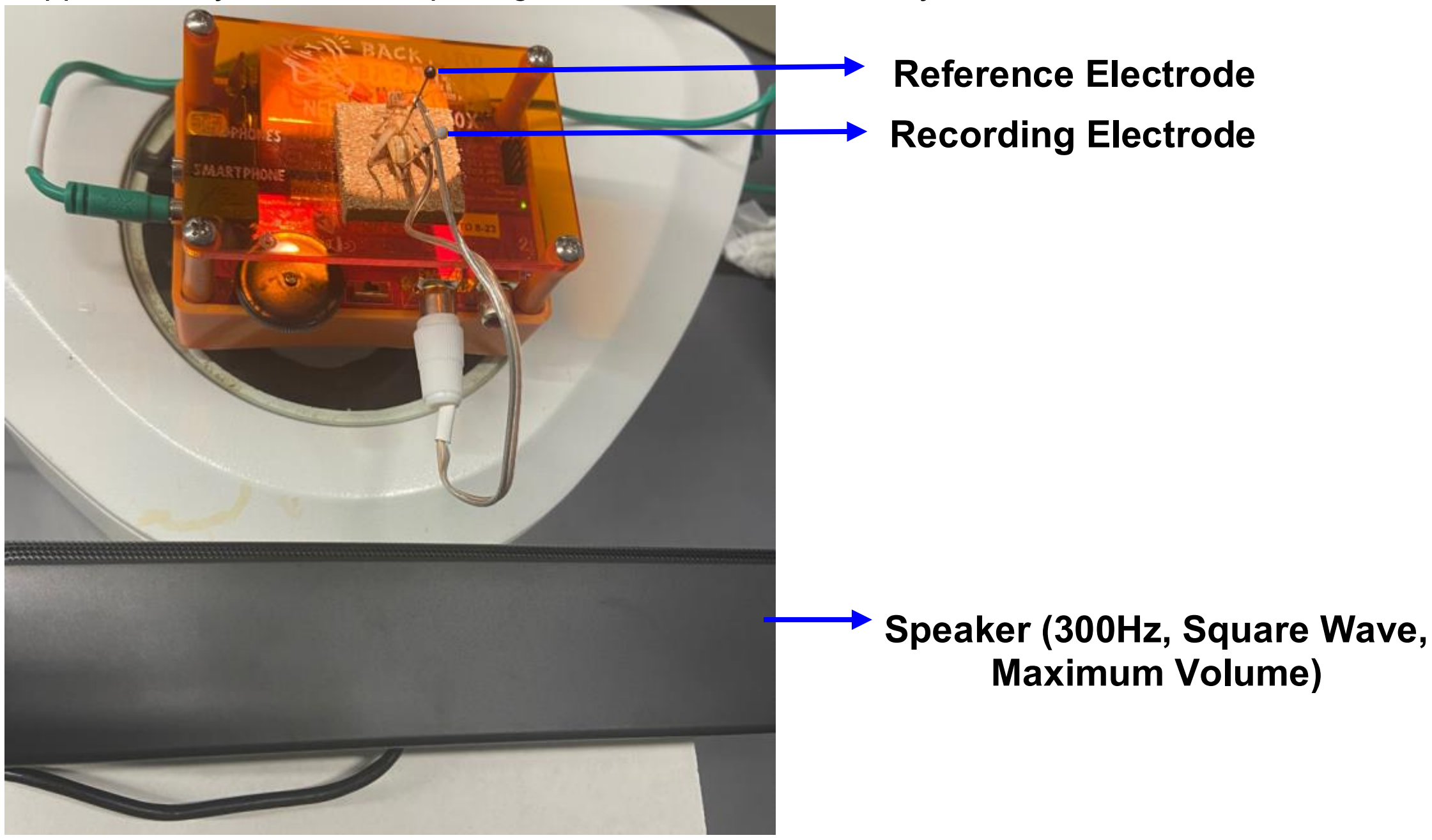

Figure 1. Cricket & Acoustic Stimulus Setup. An adult cricket is pinned to the corkboard of a SpikerBox using a reference and recording electrode. A speaker is facing the cricket's terminal abdominal ganglion, with a 300Hz square wave acoustic stimulus played at 6 and 12sec of each experimental recording.

Results:

Experiment Title:	Glutamate Treatment Piloting
Date	11/3/2023
Scientist	SA
Collaborating Scientists	NR, JM
Acquisition Highpass/Lowpass Filter Settings	160 Hz/3700 Hz; Full volume
Saved File Location	Bio302_Fall 2023 Student Data_JM_Week 8

Cricket ID	Sex	Time of anesthesia	Filename	Time of Recording	Stimulus Description	Treatment Description	Experimental Notes/Observations
01	M	3:00pm	BYB_Recording_2023-11-03_15.08.26.wav	3:08 pm	5sec no stimulus, 300Hz, square wave, max volume stimulus	No treatment	High and wide peaks, immediately after exposure to stimulus.

					at 6sec and 12sec of the recording. End with 5sec of no stimulus		
01	M	3:00pm	BYB_Recording_2023-11-03_15.12.49.wav	3:12 pm	5sec no stimulus, 300Hz, square wave, max volume stimulus at 6sec and 12sec of the recording. End with 5sec of no stimulus	1:10 (990ul of H2O, 10ul 25mM of Glutamate)	Single, small peaks. Not as wide as when it was untreated. We also saw the cricket flex
02	F	3:20pm	BYB_Recording_2023-11-03_15.28.01.wav	3:28 pm	5sec no stimulus, 300Hz, square wave, max volume stimulus at 6sec and 12sec of the recording. End with 5sec of no stimulus	No treatment	Once again, clear wide area of peaks was observed when the cricket was exposed to the stimulus.
02	F	3:20pm	BYB_Recording_2023-11-03_15.33.43.wav	3:33 pm	5sec no stimulus, 300Hz, square wave, max volume stimulus at 6sec and 12sec of the recording. End with 5sec of no stimulus	H2O	The cricket flexed, so the flexing is its response to the injection not the glutamate treatment specifically. The spikes were wide and clear, similar to the spikes we saw in the untreated cricket.
03	F	3:39pm	BYB_Recording_2023-11-03_15.47.01.wav	3:47 pm	5sec no stimulus, 300Hz, square wave, max volume stimulus at 6sec and 12sec of the recording. End with 5sec of no stimulus	No treatment	There was a strong and clear spike when given the first stimulus.
03	F	3:39pm	BYB_Recording_2023-11-03_15.50.02.wav	3:50 pm	5sec no stimulus, 300Hz, square wave, max volume stimulus at 6sec and 12sec of the recording. End with 5sec of no stimulus	1:100, (900ul H2O, 100ul 25mM glutamate)	There didn’t seem to be an inihbitory response as seen in the 1:10 dilution.

MATLAB Code: Similar to the code generated on 10_11_2023, with any changes highlighted in **yellow**:-

```
raw_data = readmatrix("No_Area_SA_Vid02_Analysis.xlsx");
for col = 2: width(raw_data)-1 %Start at column 2 to avoid the variable column,
and end with -1 to avoid background column
subtract_data(:, col) = raw_data(:,col) - raw_data(:,end);
F0(:, col) = median(subtract_data(:,col));
delta_F(:,col) = subtract_data(:,col) - F0(:,col);
ratio(:,col) = delta_F(:,col)/F0(:,col);
%Create Time Vector
time_vector= (1:length(raw_data));
time_sec= time_vector*0.116;
figure
plot(time_sec,ratio(:,col))
xlabel("Time_sec");
ylabel("deltaF/F0");
title("GCaMP5G Intensity SA03")
hold on
[pks, locs, peakwidth, peakprominence] = findpeaks(ratio(:,col), time_sec,
"MinPeakHeight", 0.2, "MinPeakProminence", 0.2);
plot(locs,pks, "o");
peakwidth(:,col)= peakwidth; %To give us a value for peakwidth
peakprominence(:,col) = peakprominence; %To give us a value for peakprominence
if col==2
title("Region 01")
elseif col==3
title("Region 02")
elseif col==4
title("Region 03")
else
  end
  end
```

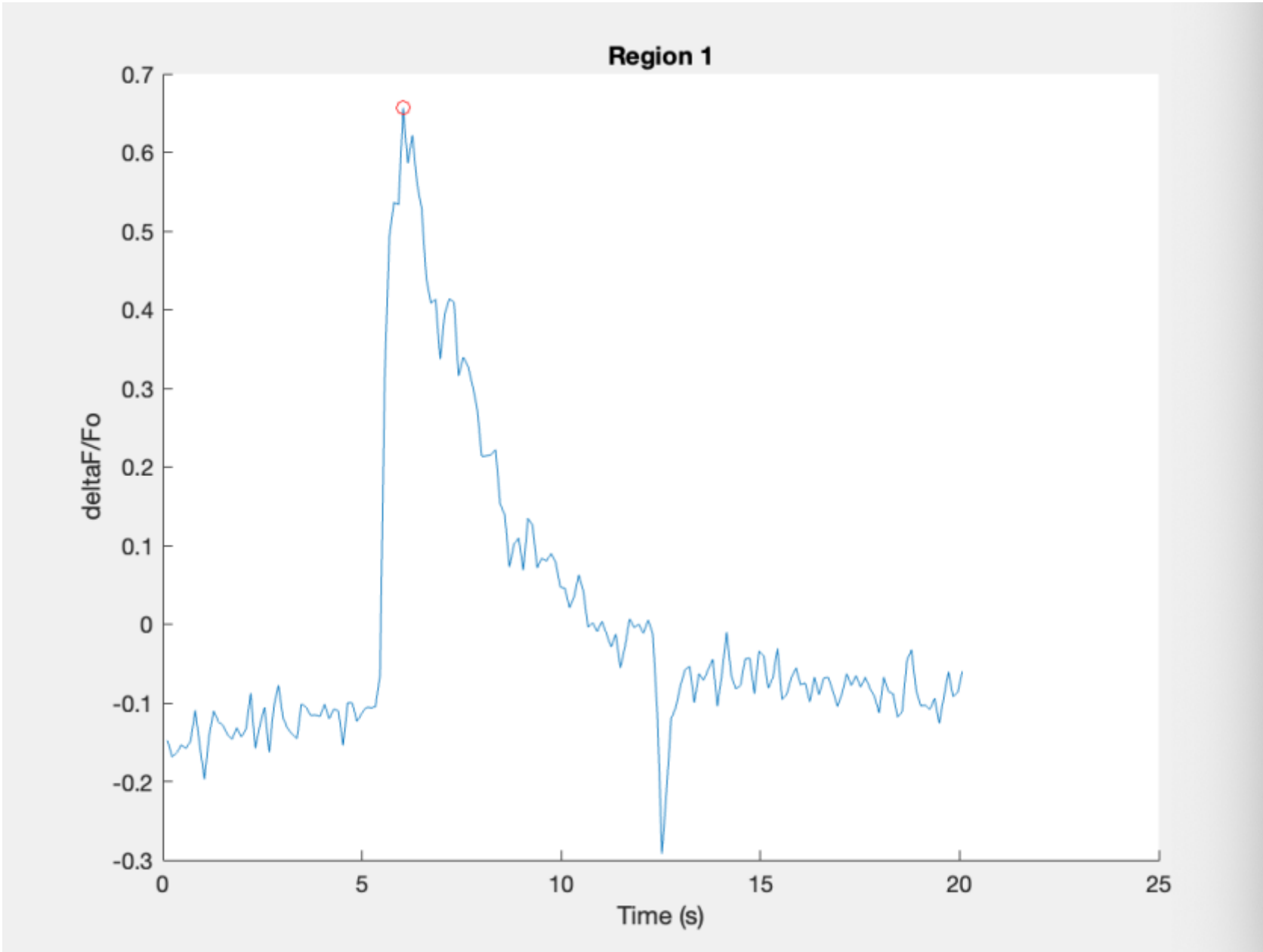

Figure 2. Change in Fluorescence Over Baseline Fluorescence Plot of Glycinergic Neurons in 5-Day Old TLF Larvae. Fluorescence of the right activated glycinergic neurons at a z-position of 2200um from 2023111_AMR_Fish01_VID01 recording. Minimum peak height and peak prominence were set at >0.2. Analysis was performed by JM using MATLAB.

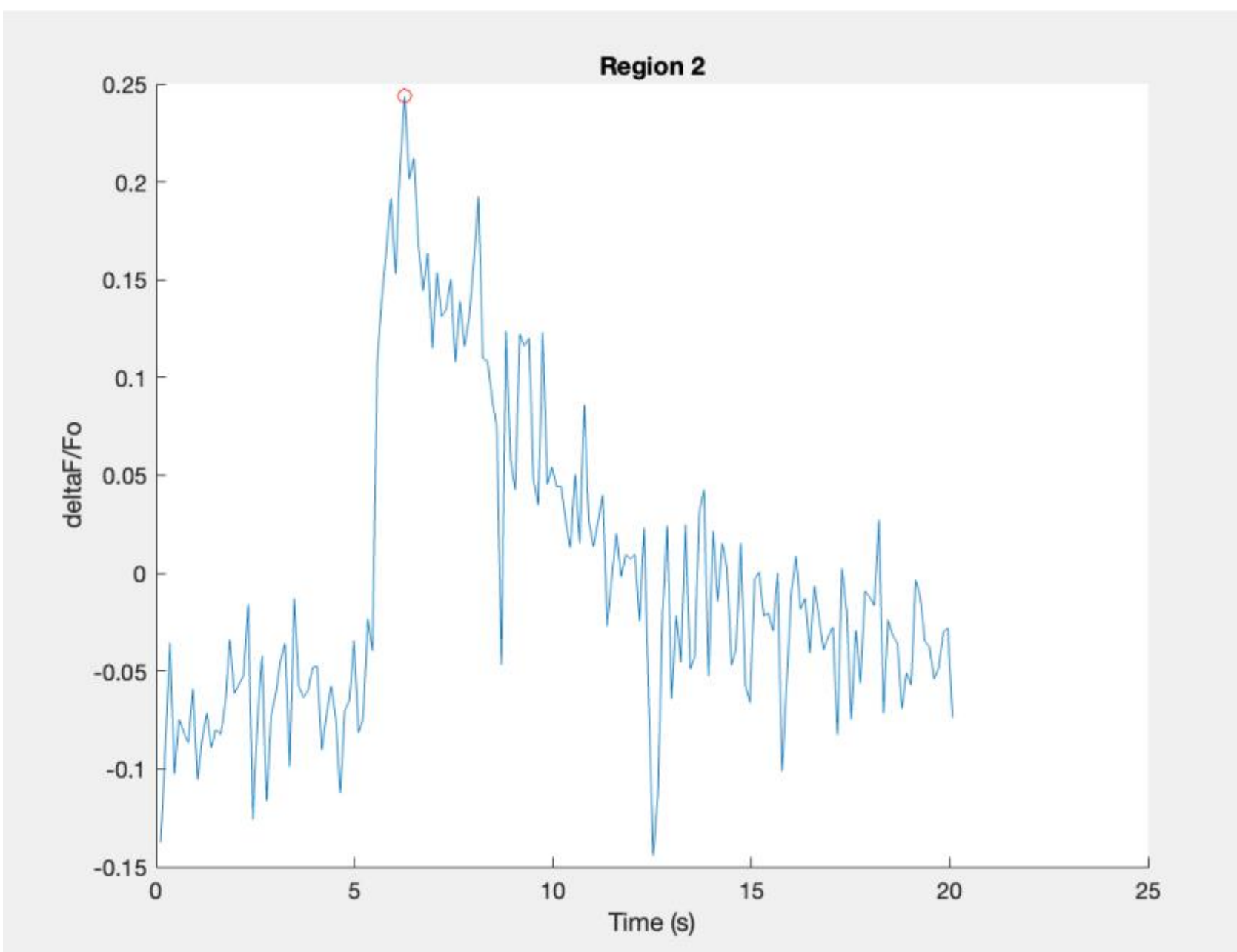

Figure 3. Change in Fluorescence Over Baseline Fluorescence Plot of Glycinergic Neurons in 5-Day Old TLF Larvae. Fluorescence of the left activated glycinergic neurons at a z-position of 2200um from 2023111_AMR_Fish01_VID01 recording. Minimum peak height and peak prominence were set at >0.2. Analysis was performed by JM using MATLAB.

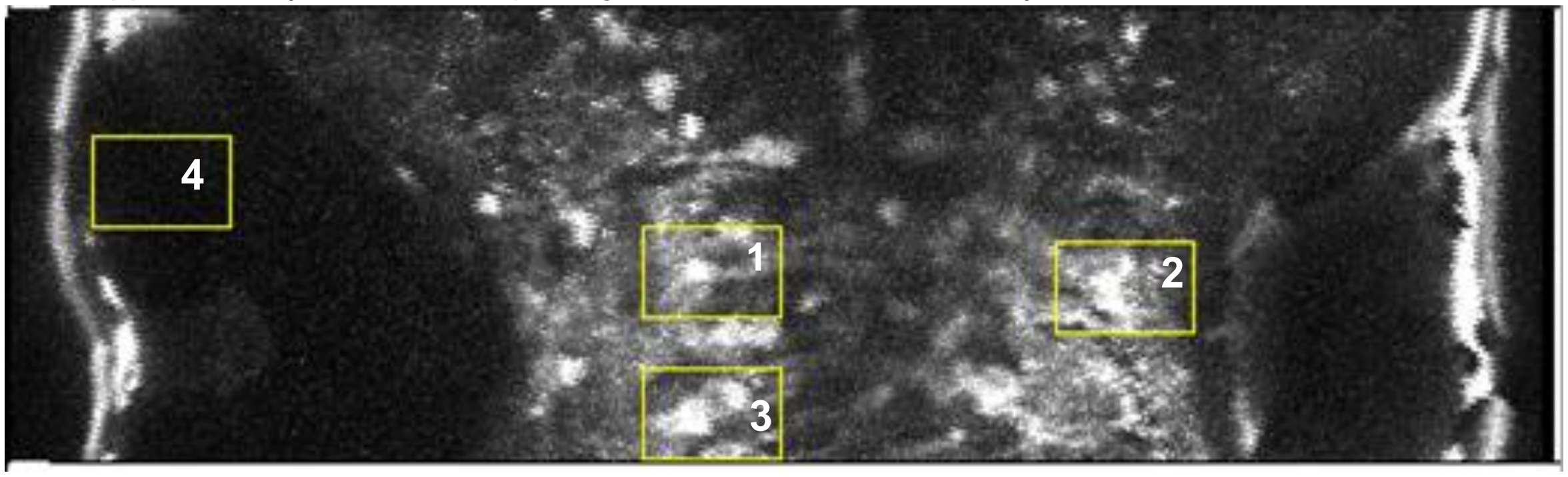

Figure 4. Adjusted Maximum Intensity of ROIs Selected at a Z-position of 2049um from 20231101_ AMR _FishSA03_VID02 . Regions 1-3 are regions with identified glycinergic neurons that respond to acoustic stimuli. The fourth region is the background with no responsive neurons used for normalization. ROI selection was done in FIJI.

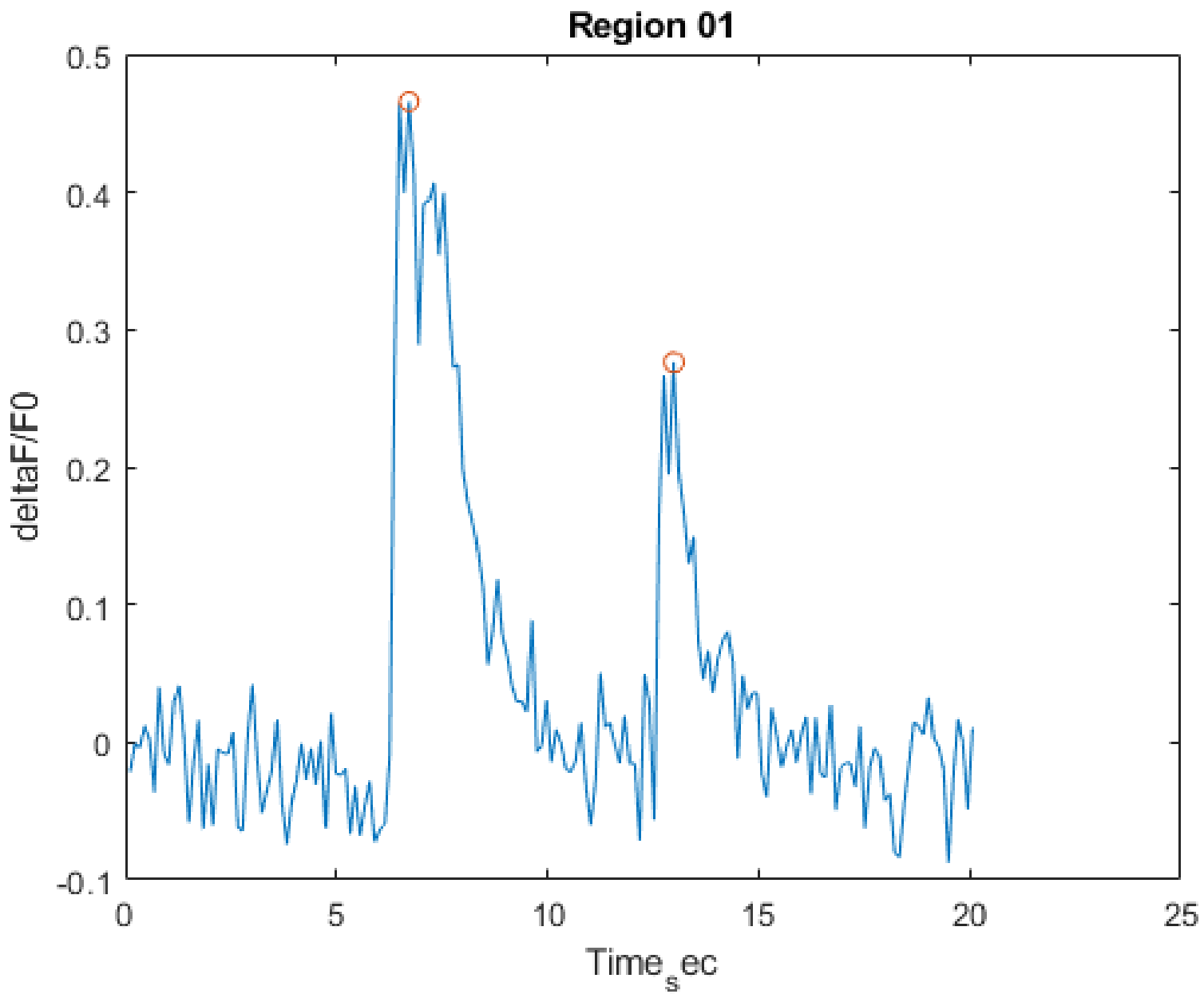

Figure 5. Change in Fluorescence Over Baseline Fluorescence Plot of Glycinergic Neurons in 5-Day Old TLF Larvae at Z-poistion of 2049um. Fluorescence of the middle activated glycinergic neurons from 20231101_ AMR _FishSA03_VID02 recording. Minimum peak height and peak prominence were set at >0.2. Analysis was performed in MATLAB.

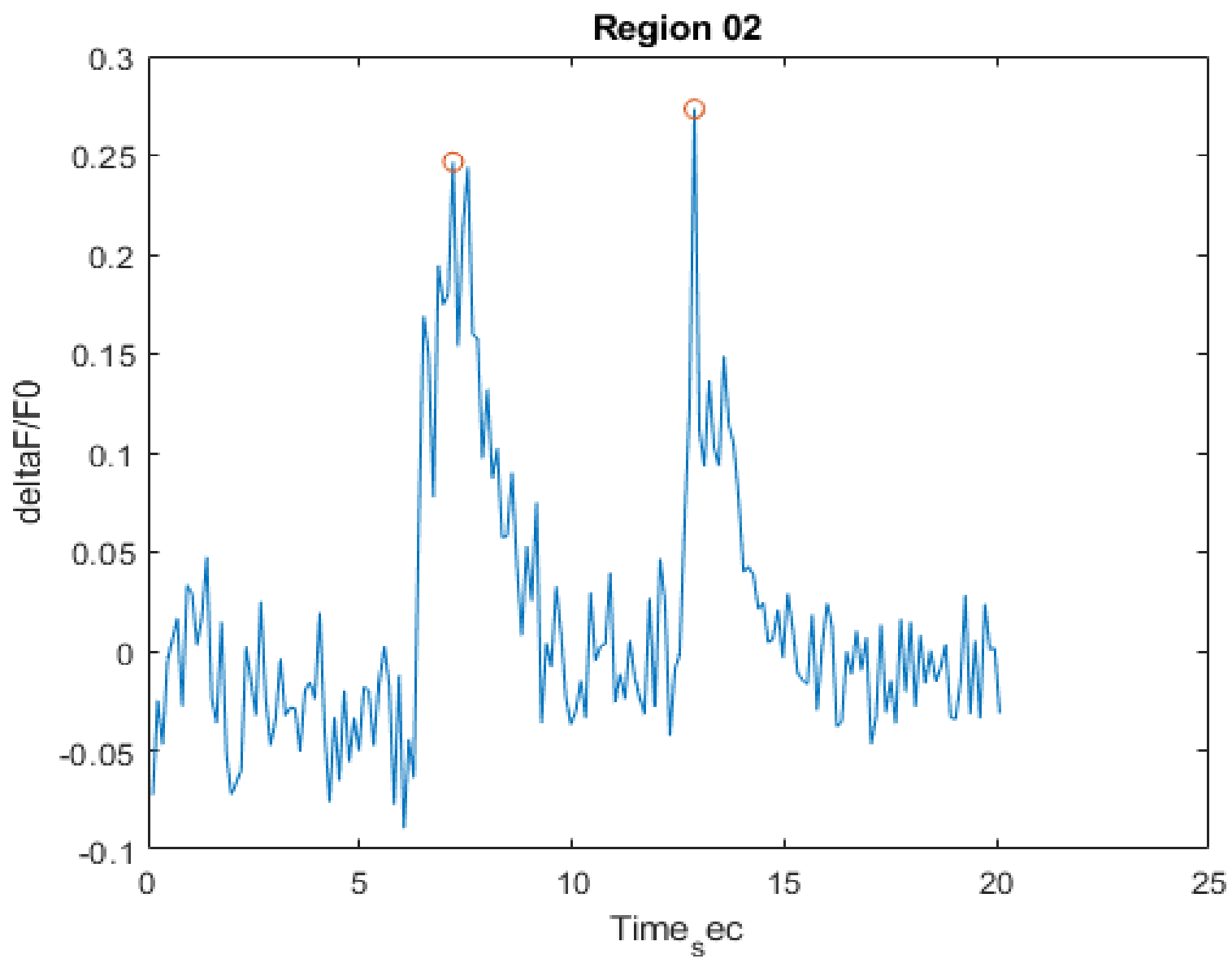

Figure 6. Change in Fluorescence Over Baseline Fluorescence Plot of Glycinergic Neurons in 5-Day Old TLF Larvae at Z-poistion of 2049um. Fluorescence of the right activated glycinergic neurons from 20231101_ AMR _FishSA03_VID02 recording. Minimum peak height and peak prominence were set at >0.2. Analysis was performed in MATLAB.

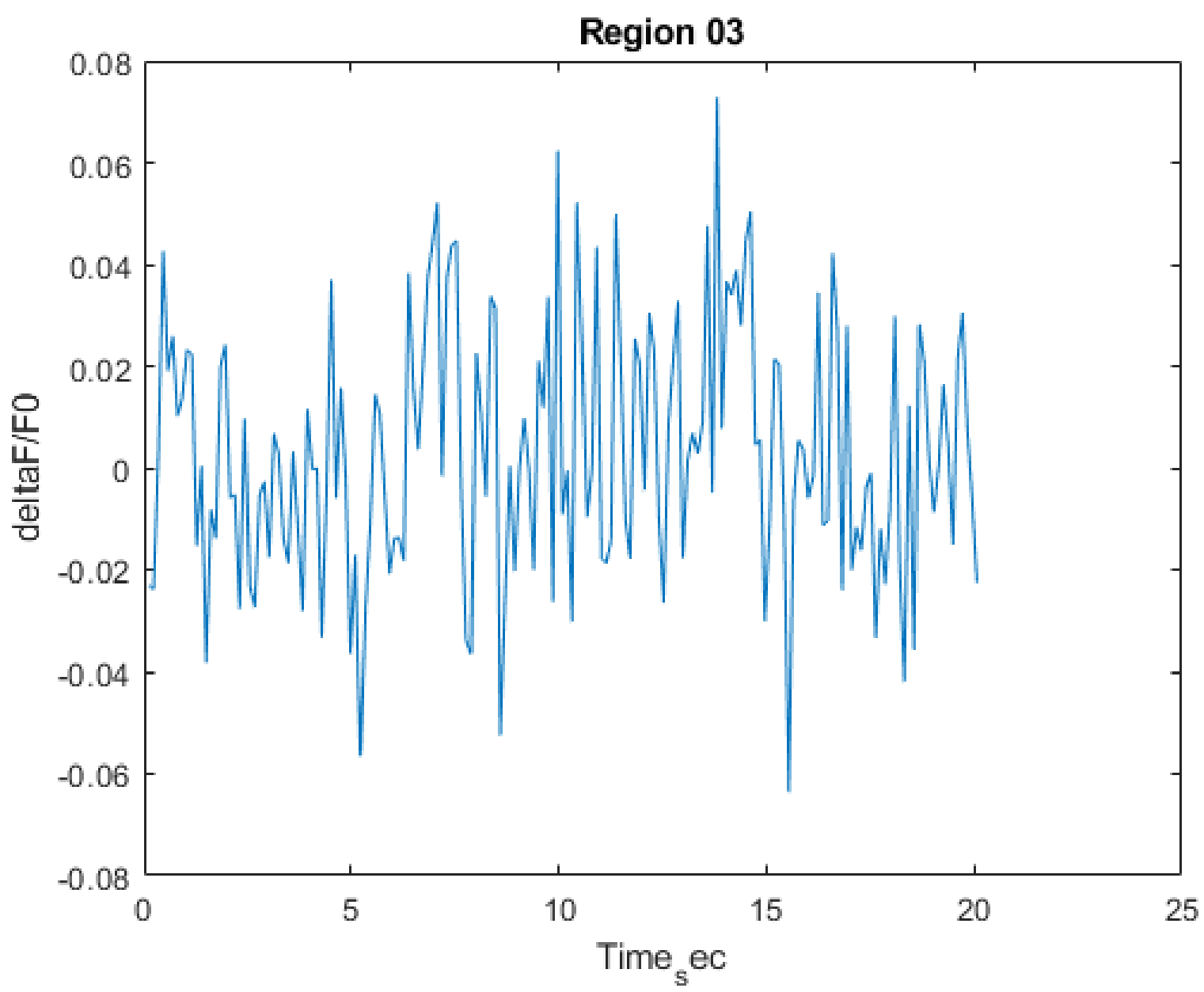

Figure 7. Change in Fluorescence Over Baseline Fluorescence Plot of Glycinergic Neurons in 5-Day Old TLF Larvae at Z-poistion of 2049um. Fluorescence of the lower, middle activated glycinergic neurons from 20231101_ AMR _FishSA03_VID02 recording. Minimum peak height and peak prominence were set at >0.2. Analysis was performed in MATLAB.

Discussion:

In today's lab, we dedicated our time to analysis of the calcium imaging recordings of the TLF larvae exposed to an acoustic stimulus, and initial piloting of glutamate treatment in adult crickets. In terms of our MATLAB analysis of the fluorescence imaging in TLF larvae, we found it challenging to identify specific ROIs to analyze since we are using the zebrafish line that selectively labels all glycinergic neurons. We decided to analyze different z-positions in the two different fish samples we used for our recordings yesterday, and our delta F/ F0 suggested activation of several glycinergic neurons in different regions of the hindbrain, indicated by large peaks >0.2 around the same time the

stimulus was given (**Figure 1-6**). A major limitation of our study, however, is going to be identifying the same group of responsive glycinergic neurons in all the larvae. To account for this, we may have to record each TLF larva prior to any treatments, to identify regions of glycinergic neurons that are responsive to our chosen acoustic stimulus, and then compare the activity of these glycinergic neurons in the same larva following the MK-801 or Glutamate treatment.

For our initial piloting of the glutamate treatment in the crickets, we observed an inhibitory response when the cricket was injected with a 1:10 dilution of 25mM glutamate. The non-treated and water-treated crickets showed a distinct response to our acoustic stimulus of 300Hz, square wave, where there was an immediate, sharp peak followed by a wide region of smaller peaks during the duration of the stimulus. In comparison, the glutamate treated cricket showed a much smaller initial peak, with a more narrow region of small peaks. However, this is based solely on our observation, and the recordings will have to be quantified and analyzed for root mean square, in order to determine whether or not there is a statistically significant difference between the responses of the glutamate-treated crickets and controls. Additionally, we only saw that inhibitory response in one cricket that was treated with a 1:10 dilution of the 25mM Glutamate. The cricket treated with the 1:100 dilution had a response similar to the control crickets. Therefore, we need to perform the experiment on at least three more crickets, in order to investigate whether our initial results replicate. If these results replicate, and Glutamate has an inhibitory effect in crickets, then our hypothesis based on the Kawasaki et al. study is supported. In the following lab, we will analyze the recordings generated today, perform more replicates of the glutamate treatment in crickets, and pilot MK-801 treatment in crickets.

2023-11-08: Day 3 of Group Research Project for Collection of Cricket Data with Replicate Crickets Testing MK-801 Concentrations

Purpose:

In today's lab, our goal is to perform more cricket electrophysiology readings to get replicate data to confirm or question our previous data that we collected in the previous week's lab. Our previous data suggests that glutamate may have an inhibitory effect on the cricket's escape response. If we see differences in the data, either we have procedural differences which are convoluting our results, there is just some natural variation within the crickets per treatment, or there is something else going on that we will have to think deeply about to understand. We will try to control all of the variables in the setup and in the actual data collection to ensure that we are keeping all of the parameters within our control the same so that any observable changes are strictly from the pharmacological effect. This is easier said than done however there are certain landmarks that we have decided on for specific details in our setup like the relative placement of the spiker box to the speaker, the placement of the electrodes, the time in between injections and recording, etc. Our goal is to primarily acquire some replicate data for glutamate and the vehicle with Milli-Q water, and to get novel data for MK-801 and a vehicle control with DMSO (since MK-801 is dissolved in DMSO). This is important because with the limited amount of time that we have in the lab, we need to make the most of it and be able to collect as much data as possible so that we have enough to analyze and to decide on concentrations for our final analysis. The scientific rationale behind performing the same pharmacological treatment on multiple replicate subjects is to be able to present robust, and redundant data to show that are results are reproducible for others. This has been a bit of a struggle for us with the cricket's because there seems to be a lot of variability. In the grand scheme of things, this data will help us to form some interim conclusions about the effects on the neural activity of crickets specifically for escape behavior. We will be able to see where we have questions and where we need to collect more data which will help guide our research. We are still in the early stages of our findings and are still working on optimizing our procedure especially with the setup.

Protocol:

1. Follow the [Cricket EPHYS protocol].
   Adjustments:
2. We will be using DMSO as a vehicle control
   a. Make Concentrations:
   b. From 50mM stock MK801 in DMSO
   c. Make 500uM
      i. 1mL of stock + 99mL DMSO
   d. Make 250uM
      i. 50mL of 500uM + 50mL DMSO
   e. DMSO at control 0.5ml per injection
   f. Instructions for glutamate dilutions in previous protocol
3. All other steps are the same as before

Results:

Cricket Pharmacology/EPHYS Experiment 11/8/23

Experiment Title:	Continuation of Pharmacological testing on Adult Cricket EPHYS readings
Date	10/27/23
Scientist	JM
Collaborating Scientists	NR, SA
Acquisition Highpass/Lowpass Filter Settings	160 Hz/3700 Hz; partial volume
Saved File Location	JM/music/Spike Recorder/Cricket Electrophysiology Week 9

Cricket ID	Sex	Time of anesthesia	Filename	Time of Recording	Stimulus Description	Treatment Description	Experimental Notes/Observations
01	F	1:26 pm	BYB_Recording_2023-11-08_13.37.23.wav	1:37 pm	6 seconds nothing, 2 seconds 300 Hz Square shape, then 6 seconds nothing twice	Untreated	Cricket spikes were relatively low during the acoustic stimulus especially compared to other groups
01	F	1:26 pm	BYB_Recording_2023-11-08_13.41.26.wav	1:39pm	6 seconds nothing, 2 seconds 300 Hz Square shape, then 6 seconds nothing twice	Treated with 0.5 ml DMSO	During treatment a lot of spikes direclty following the injection.
02	M	1:45 pm	BYB_Recording_2023-11-08_13.53.13.wav	1:53 pm	6 seconds nothing, 2 seconds 300 Hz Square shape, then 6 seconds nothing twice	untreated	The initial spike was not as strong as we have seen in other untreated crickets
02	M	1:45 pm	BYB_Recording_2023-11-08_13.54.46.wav	1:54 pm	6 seconds nothing, 2 seconds 300 Hz Square shape, then 6 seconds nothing twice	untreated	Initila spike was not as high as in other untreated crickets again
02	M	1:45 pm	BYB_Recording_2023-11-08_13.59.32.wav	1:59 pm	6 seconds nothing, 2 seconds 300 Hz Square shape, then 6 seconds nothing twice	Treated with 0.5 ml of 500μM MK-801	The recording ran for 1.5 minutes since we forgot to end the recording<br>We forgot to stop this recording
03	F	2:03 pm	BYB_Recording_2023-11-08_14.09.07.wav	2:09 pm	6 seconds nothing, 2	untreated	Spiking seemed to be much larger than the other tests so

					seconds 300 Hz Square shape, then 6 seconds nothing twice		the cricket may be more responsive. The cricket also was displaying more spontaneous spiking
03	F	2:03 pm	BYB_Recording_2023-11-08_14.14.45.wav	2:14 pm	6 seconds nothing, 2 seconds 300 Hz Square shape, then 6 seconds nothing twice	Treated with 0.5 ml of 250 μM MK-801	Cricket had a much smaller response in the spiking which may indicate an imhitoru response.
04	F	2:19 pm	BYB_Recording_2023-11-08_14.25.20.wav	2:25 pm	6 seconds nothing, 2 seconds 300 Hz Square shape, then 6 seconds nothing twice	Untreated	We used a lower volume for the speaker box to see if the cricket was being habituated which seemed to produce better spikes
04	F	2:19 pm	BYB_Recording_2023-11-08_14.30.05.wav	2:30 pm	6 seconds nothing, 2 seconds 300 Hz Square shape, then 6 seconds nothing twice	Treated with 0.5ml of 250 μM MK-801 time of treatment; 2:27 pm	Responsive to tactile stimuli but not acoustic stimuli. Was moving around but spiking was spontaneous movement<br>We forgot to stop this recording
05	M	2:38 pm	BYB_Recording_2023-11-08_14.44.22.wav	2:44 pm	6 seconds nothing, 2 seconds 300 Hz Square shape, then 6 seconds nothing twice	untreated	Cricket spiking was much higher at the initial spike and then the spikes decayed over the duration of the stimulus.
05	M	2:38 pm	BYB_Recording_2023-11-08_14.49.20.wav	2:49 pm	6 seconds nothing, 2 seconds 300 Hz Square shape, then 6 seconds nothing twice	Treated with 1:10 of the 25mM stock solution of glutamate	Lots of spontaneous baseline spiking. Increased spiking at the stimulus
06	M	2:53 pm	BYB_Recording_2023-11-08_14.58.47.wav	2:58 pm	6 seconds nothing, 2 seconds 300 Hz Square shape, then 6 seconds nothing twice	Untreated	High initial peak but there was not a consistent height over the duration of the stimulus which is what we usually see
06	M	2:53 pm	BYB_Recording_2023-11-08_15.03.12.wav	3:03 pm	6 seconds nothing, 2 seconds 300 Hz Square shape, then 6 seconds nothing twice	Treated with 1:10 of the 25mM stock solution of glutamate, treatment at 3:01 pm	Didnt appear to be much of a change for this cricket from the untreated
07	F	3:09 pm	BYB_Recording_2023-11-08_15.15.13.wav	3:15 pm	6 seconds nothing, 2 seconds 300 Hz Square shape, then 6 seconds nothing twice	untreated	Almost no activity for the stimuli so we chose not to do a treatment recording, we forgot to stop this recording so it ran long
08	M	3:18 pm	BYB_Recording_2023-11-08_15.25.43.wav	3:25 pm	6 seconds nothing, 2 seconds 300 Hz Square shape, then 6 seconds	untreated	Good spikes during the stimuli, high initial peak and somewhat of a gradual decay time

					nothing twice		
08	M	3:18 pm	BYB_Recording_2023-11-08_15.28.34.wav	3:28 pm	6 seconds nothing, 2 seconds 300 Hz Square shape, then 6 seconds nothing twice	Treated with 50 µl of Milli-Q water	Seemed to have the same spiking as the untreated test. The cricket had some spontaneous spiking directly after the first stimulus.
09	F	3:31 pm	BYB_Recording_2023-11-08_15.39.27.wav	3:39 pm	6 seconds nothing, 2 seconds 300 Hz Square shape, then 6 seconds nothing twice	untreated	The spikes seemed to have a moderate intensity for the stimulus initially but had a good area of spiking for the entire duration of the stimulus.
09	F	3:31 pm	BYB_Recording_2023-11-08_15.42.22.wav	3:42 pm	6 seconds nothing, 2 seconds 300 Hz Square shape, then 6 seconds nothing twice	Treated with a small amount of 250µM MK-801	The spiking seemed to be the same as the untreated if any thing, slightly smaller
10	M	3:46 pm	BYB_Recording_2023-11-08_15.53.09.wav	3:51 pm	6 seconds nothing, 2 seconds 300 Hz Square shape, then 6 seconds nothing twice	untreated	The second stimulus had better spiking than the first.
10	M	3:46 pm	BYB_Recording_2023-11-08_15.57.08.wav	3:57 pm	6 seconds nothing, 2 seconds 300 Hz Square shape, then 6 seconds nothing twice	Treated with DMSO	There seemed to be an inhibitory response, the spikes were almost invisible.

The recordings that are highlighted are corrupted since they were not stopped on time and are upwards of several minutes long which makes them uninterpretable.

Discussion:

- In todays lab, we performed several tests on our crickets to analyze changes in electrophysiology readings before and after treatment with DMSO, MK-801 dissolved in DMSO, and a few replicates with MilliQ water and glutamate. We used our previously determined cricket ephys protocol as our guide for the procedure in lab and decided on testing concentrations of MK-801 at 500µM and 250µM. For the glutamate, we performed a 1:10 dilution of the 25mM stock solution because we only performed one test using glutamate the previous lab.
- One conclusion that we made is that we had to scrap our preliminary conclusion on the inhibitory effect of glutamate because with the two replicate tests that we performed today, we saw variable results that seemed to not be consistently inhibitory. Therefore, we will have to continue to run trials and also confirm our eye tests using MATLAB to make sure that we are not being biased in our interpretation of the results. We also were starting to conclude that the DMSO was generating an inhibitory effect on the trials that we ran today since there was consistently less activity from before and after treatment with DMSO. This is concerning because MK-801 is dissolved in DMSO so we have to use DMSO for that stock solution. One thing that we talked about with the TA's was to dilute down the DMSO for the vehicle control as much as possible to see if we could mitigate the inhibitory response as much as possible.

- A challenge that we faced several times today was overall inconsistency with the crickets. We performed many trials (20 total recordings) and from cricket to cricket there was still little to no consistency even with baseline untreated readings. In some crickets, we observed a large initial spike and then no residual spike following, in some cases we saw a large to moderate initial spike and some moderate residual spikes for the duration of the stimulus, and in some we saw a mild to moderate initial spike followed by moderate to mild residual spiking. We are a bit lost as to the best way to deal with this issue because it almost seems like luck of the draw when we mount the crickets since everything else seems to be the same including the exact same volume and frequency for the audio stimulus. Another challenge was that several times we lost track of how long we had the anesthesia going as we were trying to many things at once so inevitably, we messed up the timing of anesthesia and went over the 90 second time. In the future, we will make sure to have the timing be a priority regardless of whatever else is going on so that we can keep our protocol consistent. Another point that we talked about is that we always had a different person doing a different component of the setup of the experiment, so we decided on assigning roles for each step so that there is consistency.
- Our next steps are to go into MATLAB and confirm that what we are seeing in the spike recordings were accurate. For example, for three total glutamate readings and corresponding water vehicle recordings, we should look at them side by side and analyze whether there was a consistent trend in the treated compared to the untreated crickets. The trouble with this is that we dont really know how much of a change we would need to see to consider it a significant inhibitory or excitatory effect since there is also some variability with our readings in general.
- Based on the paper that we read about acoustic startle habituation, we know that there are glycinergic and NMDA receptors related to the Mauthner neuron in the fish's fast escape startle circuit. Our plan to find out this role is to select a good z-position based on the preliminary confocal data that we collected in the previous week, and perform assays using our protocol for pharmacological treatments to the fish using MK-801 and glutamate.

Notes:

- 1ml 99ml DMSO
- 1:1 dilution in DMSO to make 250μM
- Confirm with others notebooks

2023-12-01: Testing the Effect of MK-801 & Glutamate Treatment on Mauthner Neurons

Purpose:
Since our attempt to look at glycinergic neurons in the zebrafish larvae was unsuccessful due to the inability to detect any glycinergic neurons that are responsive to acoustic stimuli, we decided to look at neural excitability associated with startle response by looking at the Mauthner neurons instead. To look at the Mauthner neurons, we will be using the *62A:gal4; uas:gcamp5g* line to investigate the effect of Glutamate and MK-801 treatments on Mauthner excitability following exposure to an acoustic stimulus.

Protocol:
The protocol used on 11-22-2023 was used here, with the following adjustments/additions:

1. *62A:gal4; uas:gcamp5g* fish line was used instead of the *glyT2:gal4; uas:gcamp5g* line.
2. For the vehicle controls- water and DMSO- use the same volume that was used for the pharmacological drug that were dissolved in each of the vehicle control (**72ul of water** and **30ul of DMSO**)
3. We used an amplifier to expose the larvae to the acoustic stimulus of 300Hz, Square wave, with the setup shown in **Figure 1**.
4. This online frequency generator was used instead, making it easier to play a shorter duration of the acoustic stimulus,
5. Match the settings, including z-position, of each zebrafish following he treatment to the settings that were set prior to treatment.
6. Maximum volume was **NOT** used (**Figure 2**) to minimize the shaking caused by the amplifier.

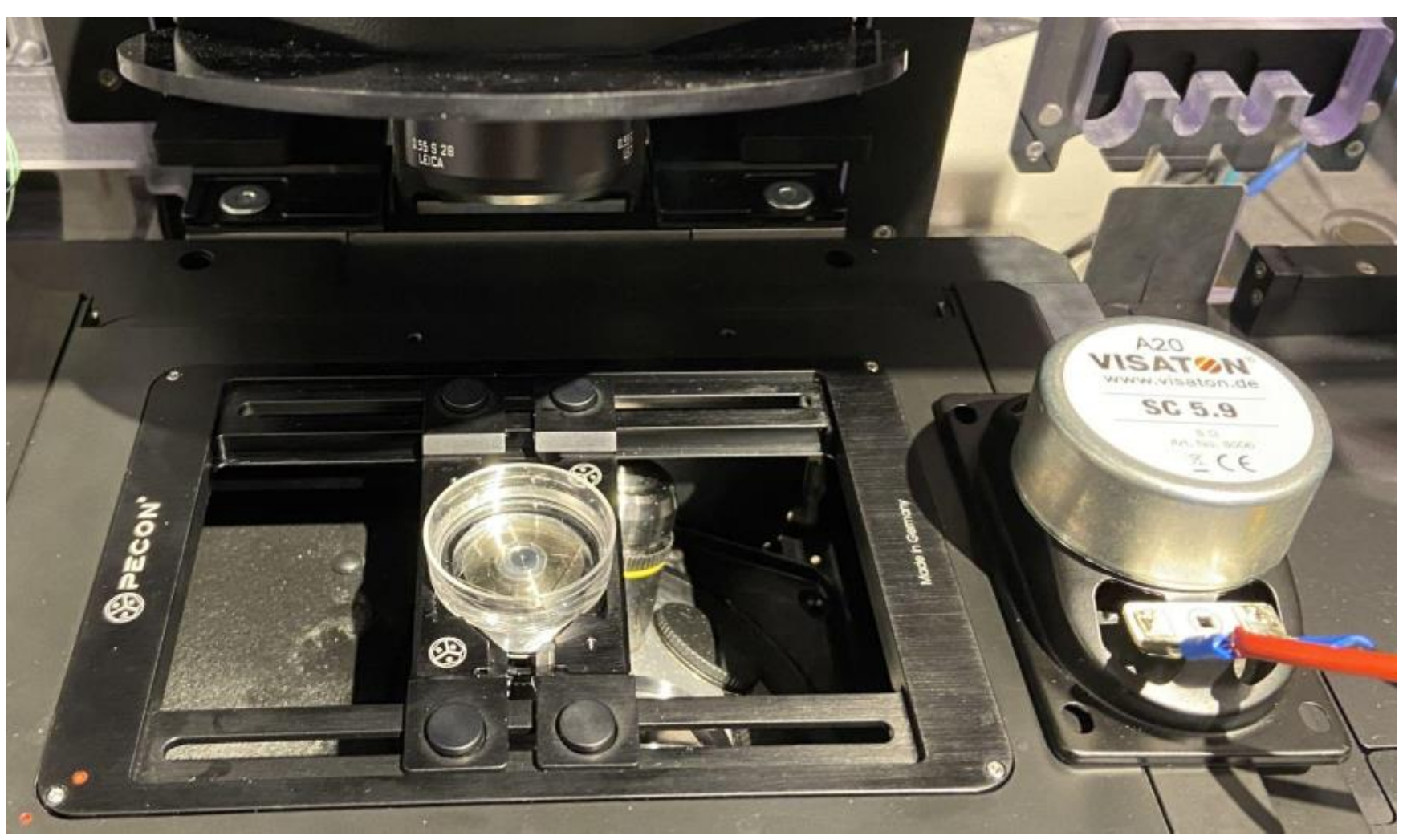

Figure 1. Amplifier Setup on the Confocal Microscope Stage

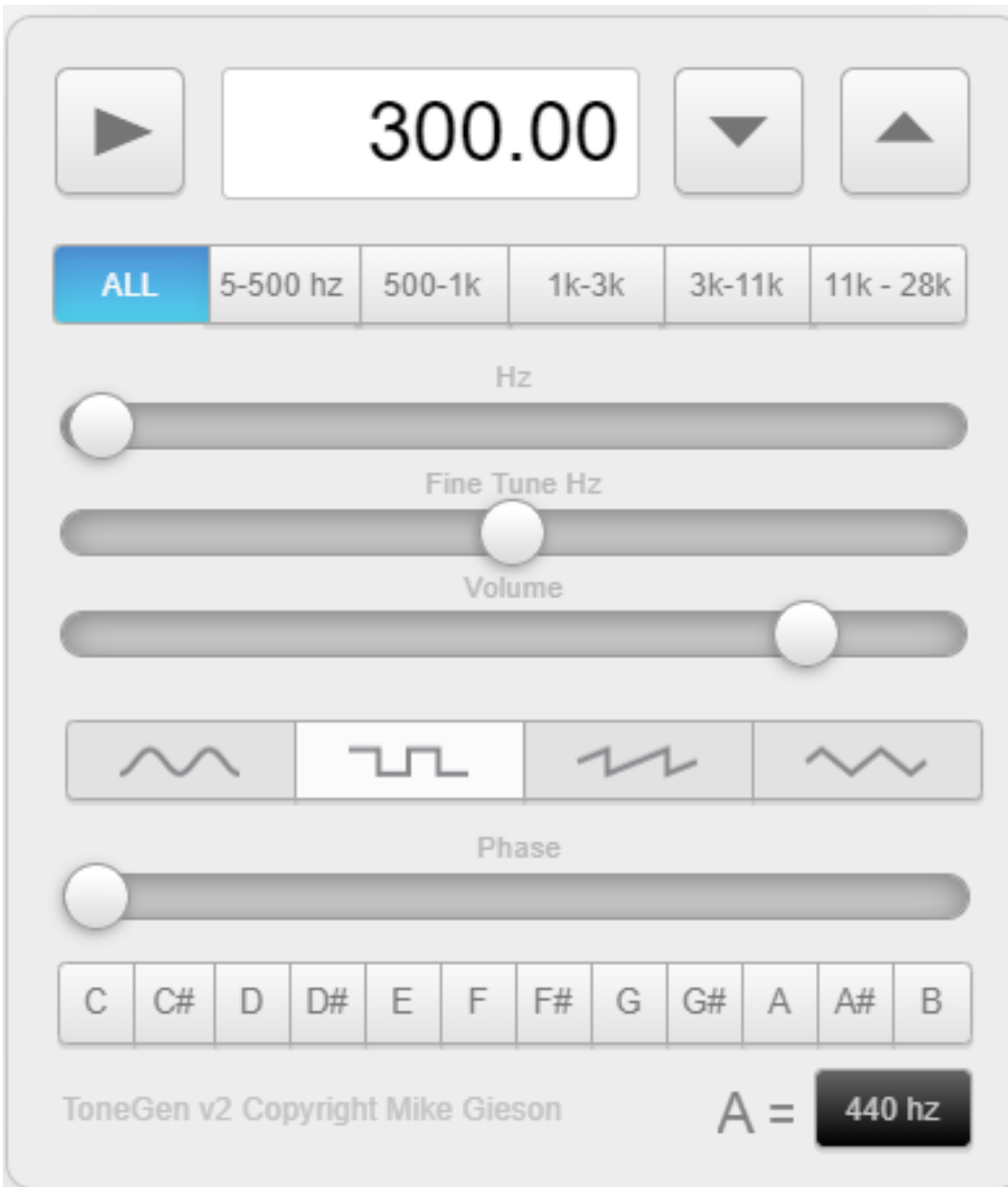

Figure 2. Settings on the Online Frequency Generator used to produce the Acoustic Stimulus.

Results:

Table 1. Mounting Trials of 7-Day Old Zebrafish Larvae

Experiment Title:	Mounting Zebrafish in Agarose	
Date	12/1/2023	
Scientist	SA	
Collaborating Scientists	Bio302 Class	
Trial #	Mount Description	Experimental Notes/Observations
01	Dorsal Mount	- Agarose hardened before I could get it to the bottom of the plate, so the head is a little more lifted than the rest of the body.
02	Dorsal Mount	- Good mount, but slightly tilted.
03	Dorsal Mount	- Good mount
04	Dorsal Mount	- Good Mount

Table 2. Calcium Imaging of Mauthner Neurons in 7-Day Old Zebrafish.

**File Name** (YYMMDD_INIT_Fish_Position_IMG##)	**Sample Info** (genetic BG, age, treatment, etc)	**Imaging Conditions** (microscope, objective, laser settings, framerate, etc)	**Comments/Observations**
20231201_AMR_JMFish03_Vid01	*62A:gal4; uas:gcamp5g*<br><br>*7dpf fish, PTU treated*	*confocal*<br>*20x objective*<br>*19.8% laser power*<br>*512x64*<br>*19.44fps*<br>Pinhole*: 5.00AU*<br>*Z-position: 1878.43*	For this mount, the fish is slightly tilted, so the left Mauthner is a little lighter. When we gave the stimulus, there was activation at the left lateral dendrite.
20231201_AMR_JMFish03_Vid02	*62A:gal4; uas:gcamp5g*<br><br>*7dpf fish, PTU treated*	*confocal*<br>*20x objective*<br>*19.8% laser power*<br>*512x64*<br>*19.44fps*<br>Pinhole*: 5.00AU*<br>*Z-position: 1878.43*	We tried using 1000 Hz instead of 300Hz, but the fish did not respond to it. Since we saw a better response to 300 Hz, we decided to stay consistent with that.
20231201_AMR_JMFish03_Vid03	*62A:gal4; uas:gcamp5g*<br><br>*7dpf fish, PTU treated*	*confocal*<br>*20x objective*<br>*19.8% laser power*<br>*512x64*<br>*19.44fps*<br>Pinhole*: 5.00AU*<br>*Z-position: 1858.70*	The amplifier completely moved the Mauthner neurons out of frame.
20231201_AMR_JMFish03_Vid04	*62A:gal4; uas:gcamp5g*<br><br>*7dpf fish, PTU treated*	*confocal*<br>*20x objective*<br>*19.8% laser power*<br>*512x64*<br>*19.44fps*<br>Pinhole*: 5.00AU*<br>*Z-position: 1883.69*	Both lateral dendrites fired, but the amplifier made the recording shake when we played the stimulus. This was after moving the amplifier a little farther away from the stage, and lowering the volume a little bit.
20231201_AMR_JMFish03_Vid05	*62A:gal4; uas:gcamp5g*<br><br>*7dpf fish, PTU treated*	*confocal*<br>*20x objective*<br>*19.8% laser power*<br>*512x64*<br>*19.44fps*<br>Pinhole*: 5.00AU*<br>*Z-position: 1883.69*	This time, we moved the amplifier even farther away. Both lateral dendrites and the left cell body fluoresced. There was a little bit of shaking so the volume was lowered a little more.
20231201_AMR_JMFish03_Vid06	*62A:gal4; uas:gcamp5g*<br><br>*7dpf fish, PTU treated*	*confocal*<br>*20x objective*<br>*19.8% laser power*<br>*512x64*<br>*19.44fps*<br>Pinhole*: 5.00AU*<br>*Z-position:1883.69*	The volume was lowered a little bit more here, and there was still slight shaking but that is probably in very few frames, so we will still be able to analyze it. Both neurons responded, with both somas and lateral dendrites fluoresced.
20231201_AMR_NRFish01_Vid01	*62A:gal4; uas:gcamp5g*	*confocal*<br>*20x objective*	The recording shook a little, but overall we were able to see clear activation of the

**File Name** (YYMMDD_INIT_Fish_Position_IMG##)	**Sample Info** (genetic BG, age, treatment, etc)	**Imaging Conditions** (microscope, objective, laser settings, framerate, etc)	**Comments/Observations**
	7dpf fish, PTU treated	*19.45% laser power*<br>*512x64*<br>*19.44fps*<br>Pinhole*: 5.00AU*<br>*Z-position:1991.93*	lateral dendrite. Only the right Mauthner neuron was labeled in this fish.
20231201_AMR_JMFish03_Vid07	*62A:gal4; uas:gcamp5g*<br><br>*7dpf fish, PTU treated, 600uM Glutamate*	*confocal*<br>*20x objective*<br>*19.45% laser power*<br>*512x64*<br>*19.44fps*<br>Pinhole*: 5.00AU*<br>*Z-position:1898.76*	15 min following Glutamate treatment, we saw a clear response to our acoustic stimulus with clear firing in the soma of both neurons and the lateral dendrite of the left Mauthner. However, the lateral dendrite of the right soma was not observed which could be due to a slightly different z-position than we had prior to treating it with Glutamate.
20231201_AMR_JMFish03_Vid08	*62A:gal4; uas:gcamp5g*<br><br>*7dpf fish, PTU treated, 600uM Glutamate*	*confocal*<br>*20x objective*<br>*19.45% laser power*<br>*512x64*<br>*19.44fps*<br>Pinhole*: 5.00AU*<br>*Z-position:1894.21*	This was recorded 20 min following Glutamate treatment, and the z-position was adjusted to better visualize the lateral dendrite of the right Mauthner. Firing in both somas and lateral dendrites of both Mauther neurons.
20231201_AMR_SAFish03_Vid01	*62A:gal4; uas:gcamp5g*<br><br>*7dpf fish, PTU treated,*	*confocal*<br>*20x objective*<br>*19.45% laser power*<br>*512x64*<br>*19.44fps*<br>Pinhole*: 5.00AU*<br>*Z-position: 2192.50*	The first stimulus changed the frame, but there was clear firing in the lateral dendrite of the right neuron. The left neuron is dim because of the tilted mount.
20231201_AMR_SAFish03_Vid02	*62A:gal4; uas:gcamp5g*<br><br>*7dpf fish, PTU treated,*	*confocal*<br>*20x objective*<br>*19.45% laser power*<br>*512x64*<br>*19.44fps*<br>Pinhole*: 5.00AU*<br>*Z-position: 1911.41*	Activation of the right soma and lateral dendrite. Slight fluorescence of the left soma, but hard to tell
20231201_AMR_NRFish01_Vid02	*62A:gal4; uas:gcamp5g*<br><br>*7dpf fish, PTU treated, 72 ul of Water*	*confocal*<br>*20x objective*<br>*19.45% laser power*<br>*512x64*<br>*19.44fps*<br>Pinhole*: 5.00AU*<br>*Z-position: 1883.50*	Fluorescence in the soma and lateral dendrite.
20231201_AMR_SAFish03_Vid03	*62A:gal4; uas:gcamp5g*<br><br>*7dpf fish, PTU treated, 500uM*	*confocal*<br>*20x objective*<br>*19.45% laser power*<br>*512x64*<br>*19.44fps*	At 15min of treatment, the Mauthner neurons were very bright, but started to fade at about 20 min. We couldn't get a good recording because the amplifier kept moving the Mauthner neurons out of

**File Name** (YYMMDD_INIT_Fish_Position_IMG##)	**Sample Info** (genetic BG, age, treatment, etc)	**Imaging Conditions** (microscope, objective, laser settings, framerate, etc)	**Comments/Observations**
	of MK-801	Pinhole*: 5.00AU*<br>*Z-position: 1919.85*	frame. We tried recording it 4x

Discussion:

Our previous attempts to investigate startle response in TLF larvae by looking at the activation of glycinergic neurons haven't been successful so far. A major issue with looking at glycinergic neurons is that we are not looking at a single neuron, Instead, we were looking at all glycinergic neurons without knowing how many are responsive to acoustic stimuli, and how many of the responsive glycinergic neurons are labeled in each of the larvae. Therefore, we decided to investigate the effect of blocking and activating NMDA receptors on startle response by looking at the Mauthner neurons, which mediate escape behavior in Zebrafish.

Since we didn't initially start with looking at the Mauthner neurons, we had to briefly troubleshoot our acoustic stimulus prior to treating the fish with MK-801 and Glutamate to ensure that the Mauthner neurons respond to the stimulus that we have chosen. We started off using the regular speaker we used when experimenting with the crickets. While we saw some activation in the lateral dendrites, it was very difficult to detect it, so we decided to try the amplifier for a better response. Using the amplifier made it very clear that the larvae were responding to the acoustic stimuli, however, we had an issue with the amplifier resulting in a change of frame during the recording, or falling off the stage. We decided to lower the volume on the computer (Figure 2) and slightly move the amplifier away from the plate with the mounted fish to avoid that issue. However, we still observed some shaking in some of the recordings, but since the Mauthner neurons did not go out of frame, we were able to clearly see the change in fluorescence.

For all the fish who had both Mauthner neurons labeled, we saw activation in the lateral dendrites and soma of both neurons. In terms of the treatment, we were able to test one replicate of Glutamate, water, and MK-801. By mere observation, we saw no significant difference in the activation of either neuron upon exposure to the acoustic stimulus following Glutamate and water treatments. For MK-801, however, we noticed that 15 minutes following the treatment, both neurons appeared a lot brighter when we visualized them on the monitor with the same image settings used prior to the treatment. Since we recorded 20 minutes following the Glutamate treatment, we waited 20 minutes to record the Mauthners following treatment with MK-801, and by then both neurons had become a lot dimmer. We had issues with the amplifier moving the neurons out of frame and falling off the stage, so we were unable to get a good

recording of the MK-801 treated fish. However, next time, we will be treating at least two more fish with MK-801 and visualizing them 15 minutes after the treatment to see if we observe the same brightness, and potentially expose them to the stimulus before the neurons become dimmer. Additionally, we will have to treat the fish with the vehicle control, DMSO, and replicate the Glutamate and water treatments at least two more times. Through these pharmacological treatments, we are hoping to get a better understanding of how the inhibition and activation of NMDA receptors impacts TLF larvae's startle response at the neurobiological level by looking at the Mauthner neurons.
