# Supplementary material for: Comparative Investigations of the Neurophysiological Mechanisms of Escape Behavior: A Course-based Undergraduate Research Experience
Source: J Undergrad Neurosci Educ. 2026 Jun 4;24(2):1–16. doi: 10.59390/001c.160661 (PMC13625905; doi:10.59390/001c.160661)
Supplement: Supplementary file 2 [file junejournal_2026_24_2_160661_340113.pdf]

Bio302 Superlab Team Research Proposal (Fall 2023)

Overview:

In this assignment, your team will write a research proposal document describing your experimental plan to pharmacologically elucidate the neural basis of escape behavior in both crickets and zebrafish. Your hypothesis-driven research should seek to connect across species and identify to what extent the neural bases of escape behaviors are similar and/or different in both of these unique systems. You will draw from primary scientific literature and your own experience so far in the course to construct a full experimental plan including a clear/concise scientific question, an informed hypothesis, a clear experimental plan including data analysis, your anticipated results and potential limitations, and interpretation of your results.

Due Dates:

Initial draft: Due **Tuesday October 24th @ 4pm** (1 per group)
Final Proposal: Due **Wednesday November 1st @ Noon** (1 per group)
Combined, this group project comprises 10% of your course grade. The draft proposal will be weighted as 1/3 of your overall assignment grade, with the final revised version weighted as 2/3.

Proposal Format:

The proposal document should be 2 pages max, single-spaced, using a font size no smaller than 11. Your figure, figure legend, and citations do not count toward the 2-page limit. Below are the guidelines for specific sections to be included in your document:

1) **Title** – Succinct, containing the overall biological concept/aim of the proposal, key words, and organisms used
2) **Keywords** – 3-6 key biological terms relevant to your proposal's goals
3) **Introduction** – State the nature of the specific, experimental problem of the proposal, adequately documented with citations for the background and rationale to support this study. Aim to cite 2-3 literature review articles and 6-8 primary research articles.
4) **Question/Hypothesis** – State your specific research question and your hypothesis.
5) **Research Plan** – Describe your experimental approach for testing your hypothesis. Clearly explain the major steps of your cricket & zebrafish experimental approach, and outline data analysis procedure (what will be measured, what will be quantified and how, what will be presented, etc.). This should include info about the proposed groups, pharmacological treatments, estimated numbers of animals and/or replicates. This section should refer directly to your Figure. Please note that your research plan is less detailed than an experimental protocol, covering only major steps, variables, and manipulations, but not the step-by-step instructions found in your lab notebook.
6) **Anticipated Results & Limitations** – Clearly articulate/explain the different possible outcomes of your experiments and analysis. Be clear about how those outcomes will be

interpreted to support or fail to support your specific hypotheses. Also, discuss any potential limitations in your experimental approach/analysis and any way by which you would overcome them if possible.

7) **Figure** – This should contain a schematic diagram of your experimental setup/plan for both crickets and zebrafish. Your Figure may have multiple panels if you wish, and must also include a written Figure Legend, beginning with a Figure Title sentence followed by a concise description of the contents of all panels of the figure. The entire Figure and Legend should fit on a single page.

8) **References** – You should include at least 2-3 relevant review articles and at least 6-8 relevant primary research articles. Please use the Nature citation style - examples of properly formatted sources for your bibliography are below:

When you need to put a reference at the end of sentence[1], you should put a superscript numeric reference identifier before the period[2].

1. Wengert, E. The title of the paper is here. *Journal A*. **25**, 145-153 (2019). ***This is an example of a single author**

2. Jain, R.A. et al., The title of the paper is here. *Journal B.* **12**, 34-45 (2020). ***This is an example for a citation that has many authors.**

Helpful tips for scientific writing

Literature: You should explore the literature around the topic/pharmacological agent that you are working on beyond the minimal journal club and background papers we have posted. This will be necessary to gain a firm grasp of the hypothesis, experimental approach and significance of your data. References and in-text citations will be essential to support your writing.

Biological names: Genus names (ex: *Danio*) begin with an uppercase letter. Species names (ex: *rerio*) are written entirely in lower case. Both genus and species names must be *italicized.* The first occurrence of a species name in a text is written in full – *Drosophila melanogaster*. In subsequent reference, the genus name can be abbreviated – *D. melanogaster*.

Gene and protein nomenclature: Gene names are italicized in lowercase (ex: *glyT2*). Protein names are written in uppercase and unitalicized (ex: GlyT2).

Research Proposal Grading Rubric

**Items**	**What is expected**	**Max Score (total 100)**	**Score Earned**
Title and Keywords	- Succinct and clear<br>- Reflects the main biological question(s) of the proposal<br>- Appropriate keywords included	5	
Introduction	- Clearly stated research problem<br>- Proper use of citations<br>- Ideas/concepts are well explained and logically ordered<br>- Discusses both experimental systems and comparative nature of research problem.<br>- Methodological approach described/defended	20	
Questions/ Hypothesis	- Clearly stated<br>- Appropriate to the research plan<br>- Attention to both generality and specificity of the project (i.e., not too specific or too general)	10	
Research Plan	- Experimental methods match the scientific question<br>- Clear description of methods & experimental groups/treatment<br>● Sufficient detail to understand approach (e.g. concentration of solution)<br>● Avoidance of unnecessary details (e.g. volume of solution)<br>● Uses both crickets and fish as experimental systems	30	
Anticipated Results & Analyses	- Clear descriptions of a variety of possible results/outcomes<br>- Description of what calculations & analyses you'll do to the functional outputs<br>- Describe what these potential results would mean about your hypothesis<br>- Relate possible results to overall biological concept of escape behavior<br>- Potential limitations articulated	10	
Figure & Legend	- Clear schematic of your experimental approach in both systems (crickets & zebrafish)<br>- Contains a Figure Legend with a clear Figure Title sentence	10	
References	- Primary literature citations (ideally 2-3 review and 6-8 primary literature articles)<br>- Proper citation format	10	
Format	- Single spaced<br>- Font size and type<br>- Two pages main text, plus 1 page figure<br>- Scientific style (biological names, etc)	5	
Overall Writing Style	- You can earn up to 5 bonus points for overall clear & coherent writing style, free of grammatical mistakes		
