# Supplementary material for: Comparative Investigations of the Neurophysiological Mechanisms of Escape Behavior: A Course-based Undergraduate Research Experience
Source: J Undergrad Neurosci Educ. 2026 Jun 4;24(2):1–16. doi: 10.59390/001c.160661 (PMC13625905; doi:10.59390/001c.160661)
Supplement: Supplementary file 3 [file junejournal_2026_24_2_160661_340114.pdf]

Comparative Investigations of the Neurophysiological Mechanisms of Escape Behavior: A Course-based Undergraduate Research Experience
Supplementary File 4. Materials List for Bio302 course.

General:
1. Laptop computer(s) running MATLAB (Mathworks).
2. Dissection Scopes with light source (Leica)
3. Heat blocks at 42ºC, for low melting point agarose aliquots in 1.7 ml microfuge tubes
4. Ice
5. General Purpose Broad-tipped Forceps (Fisher)
6. Razor Blades (Fisher)
7. Kimwipes (Fisher)
8. Confocal Microscope (Leica Stellaris 5)
9. BD Lo-Dose™ U-100 Insulin Syringes (Fisher 14-826-79)
10. Micropipetters with disposable tips
11. Samco Plastic Transfer pipets (Thermofisher 202) for handling fish larvae and stimulating cricket cercal ganglia
12. 100 mm untreated Petri Dishes for raising zebrafish embryos/larvae

Cricket Extracellular Physiology:
1. Backyard Brains Neuron Spikerbox Pro (Backyard Brains)
2. SpikeRecorder Software (Backyard Brains)
3. Live Crickets (Petco or local pet shop)

Zebrafish larvae raising & Calcium Imaging:
1. Zebrafish larvae (raised to 5-7 dpf), see Methods for transgenic lines used
2. Low Melting-Point Agarose (Sigma A9414)
3. 35 mm imaging Dish, No. 1.5 Coverslip, 10 mm Glass Diameter, Uncoated (MatTek P35G-1.5-10-C)
4. 1x E3 embryo medium [5 mM NaCl, 0.17 mM KCl, 0.33 mM $CaCl_2$, 0.33 mM $MgSO_4$, pH 7.0]
5. 1-phenyl-2-thiourea (PTU, Sigma P-7629) in 1x E3 to raise fish starting at 24 hpf
6. Fine gel loading tips and/or Dumont #5 forceps (Fine Science Tools 11251-20) for orienting larvae in agarose
7. Mini USB-speaker for acoustic stimuli (Lielongren, Amazon), or small speaker with amplifier, such as SC 5.9 OM - 8 Ohm (Visaton)
8. Pronase (Sigma 10165921001)

Pharmacological Agents (IACUC approved, only #1-5 used in our implementation):
1. (+)-MK-801 hydrogen maleate (Sigma M107), ≤500 μM
2. Fluoxetine hydrochloride (Sigma F132), ≤16.2 μM
3. (−)-Nicotine (Sigma N3876), ≤100 μM
4. L-Glutamate (Sigma G1251), ≤600 μM
5. Serotonin HCl (Sigma H9523), ≤50 μM
6. 4-aminopyridine (Sigma 275875), ≤1 mM
7. Pentylenetetrazole (Sigma P6500), ≤20 mM
8. Betamethasone 17-valerate (Sigma B0515), ≤30 μM
9. Caffeine (Nutrivitashop), ≤500 μM
10. Adenosine (Sigma A9251), ≤4 mM
11. Hexamethonium chloride (Sigma H2138), ≤100μM
12. Dopamine hydrochloride (Sigma H8502), ≤100μM
