# Supplementary material for: Comparative Investigations of the Neurophysiological Mechanisms of Escape Behavior: A Course-based Undergraduate Research Experience
Source: J Undergrad Neurosci Educ. 2026 Jun 4;24(2):1–16. doi: 10.59390/001c.160661 (PMC13625905; doi:10.59390/001c.160661)
Supplement: Supplementary file 4 [file junejournal_2026_24_2_160661_343732.pdf]

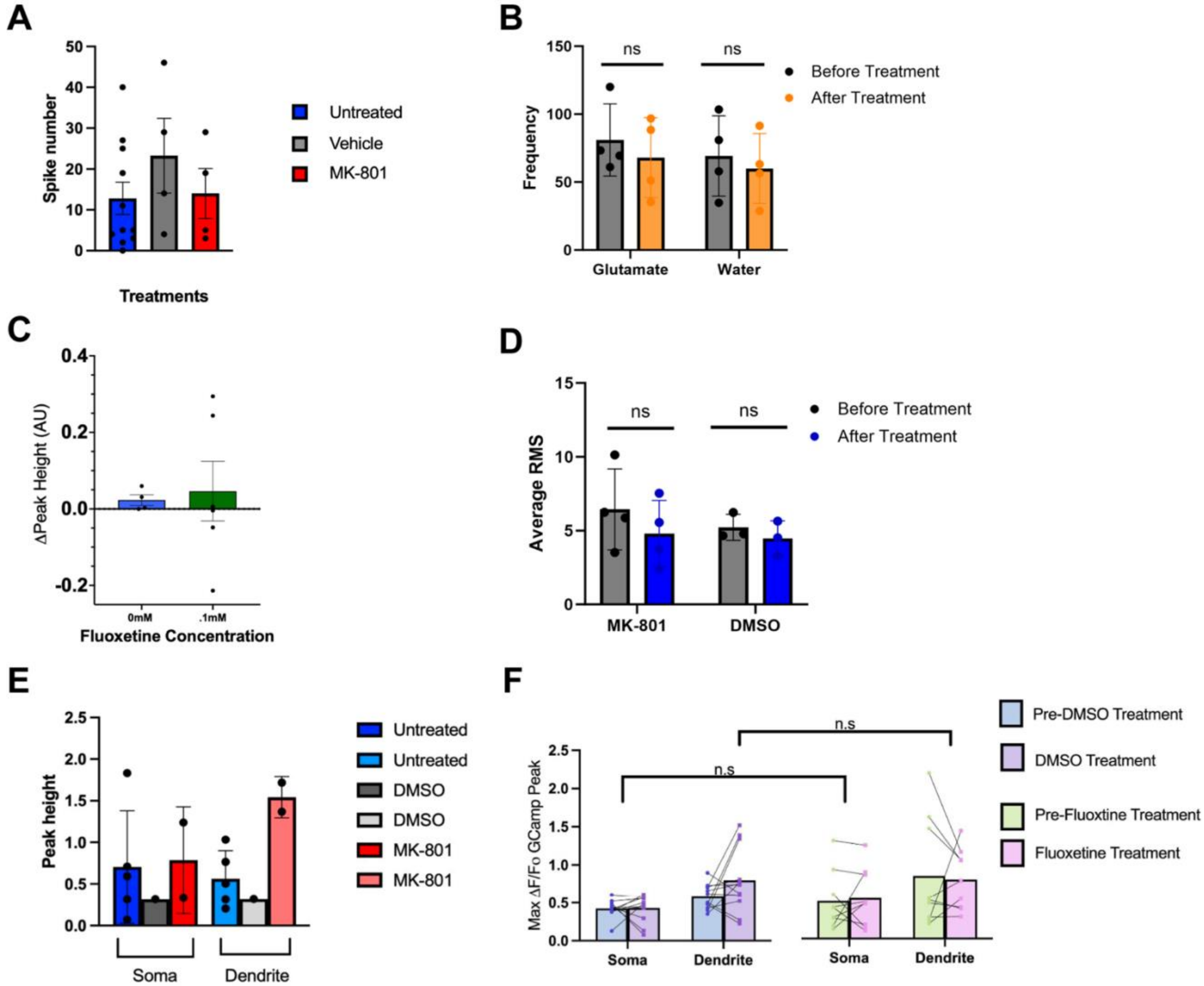

Examples of student-generated quantitative analyses, excerpted from final student presentations.

(A) Impact of acute abdominal injection of 500 µM MK-801 or vehicle (1% DMSO) on acoustically-evoked spike number in the cricket terminal abdominal ganglion. No significant difference between treatment groups, $p>0.05$, 1-way ANOVA.

(B) Impact of acute abdominal injection of 250µM Glutamate or vehicle (water) on spike frequency (Hz) in the cricket cercal ganglion. ns: $p>0.05$, paired t-test.

(C) Change in average wind-evoked spike height (AU) in the cricket cercal ganglion following acute abdominal injection of 0.1mM Fluoxetine or vehicle (1% DMSO). No significant difference between treatment groups $p>0.05$, unpaired t-test.

(D) Impact of acute abdominal injection of 500 µM MK-801 or vehicle (1% DMSO) on calculated Root-Mean-Squared (RMS) of spiking in the cricket cercal ganglion, averaged from 2 stimuli. ns: $p>0.05$, paired t-test.

(E) Impact of 20-minute 500 µM MK-801 or vehicle (1% DMSO) treatment on acoustically-evoked peak calcium fluctuation ($\Delta F/F_o$) in the soma and lateral dendrite of Mauthner neurons expressing GCaMP5G in 6 dpf zebrafish larvae. No significant difference detected between treatment groups in the soma or in the lateral dendrites $p>0.05$, 1-way ANOVAs.

(F) Impact of 20-minute 100µM Fluoxetine or vehicle (1% DMSO) treatment on acoustically-evoked peak calcium fluctuation ($\Delta F/F_o$) in the soma or lateral dendrite of the Mauthner neuron expressing GCaMP5G in 6 dpf zebrafish larvae. No significant difference between treatment groups, $p>0.05$, unpaired t-test of pre-post differences.
