# Supplementary material for: Comparative Investigations of the Neurophysiological Mechanisms of Escape Behavior: A Course-based Undergraduate Research Experience
Source: J Undergrad Neurosci Educ. 2026 Jun 4;24(2):1–16. doi: 10.59390/001c.160661 (PMC13625905; doi:10.59390/001c.160661)
Supplement: Supplementary file 5 [file junejournal_2026_24_2_160661_340111.docx]

Digital Lab Notebook:
[Danny O’Rerio ’25]

**Bio302 Superlab Fall 2023, Haverford College**

# Table of Contents

**Table of Contents	1**

**Maintaining this lab notebook	2**

**YYYY-MM-DD: Entry Template (Replace w/Experiment Title)	4**

**Imaging Table Template	5**

**Cricket Experiment Template	6**

**Q3 Lab Safety Form	7**

# Maintaining this lab notebook

1. ALL experiments and data must be recorded in your lab notebook. This includes computer-based “experiments” such as installing Matlab, ImageJ/FIJI, analyzing Excel-based data, creating Excel-based analysis pipelines, team discussion for research projects, etc.
2. For each new experiment, first copy the Entry Template below, then go to the end of the document, **Insert>Break>Section break (next page) to get to a new page**, and then paste the Template. **Every experiment must have a Purpose, Protocol, Results, and Discussion,** even inconclusive or abandoned experiments. Computer-based work, design, and analyses are also lab work, and should be appropriately detailed in your notebook.
3. Adjust the experiment start date and make a concise and descriptive title. This text must be in the modified **“Heading 1” format (Red, Underlined)** which will show up in the Outline tab for easy searching. (for example: “Analysis of spike frequency in cricket cercal ganglia treated with SSRI’s” rather than just “Matlab Analysis 2”)
4. Fill in a brief Purpose for the experiment. This should be **specific to the experiment**/steps at hand, and also **connect to the broader goal of the project**.
5. Paste in or link to your protocol. To link to an existing protocol on Drive, go to that protocol file, click on the blue “Share” button in the top right corner, then click the green “Get Shareable Link” icon in the right corner of the window that pops up to copy the location. Then return to your lab notebook entry and Insert a link, pasting your copied location into the “Link” field and typing the protocol name into the “Text” field. If you adjust the protocol you should indicate any and all deviations from the linked protocol, though it’s probably better/easier to copy/paste the protocol into your lab notebook and make the changes directly there.
6. **Fill in your data as you collect it.** For digital data, you must enter the complete name of the data file, and hyperlink to it if it is online. If not hyperlinked, state the location/folder where the data file can be found. You can paste in screenshots/phone pics of example analyzed video images, excel graphs, etc, but the location of the RAW data/digital files must also be stated/linked.
7. **Every Superlab day where you do work should have its own entry!** If an experiment spills onto the next day with just a small amount of followup, insert a new line with the new date in bold **[YYYY-MM-DD]** before you add new info, so it’s easy to see when each part was performed & entered. You can easily link back to a previous experiment by clicking “Link” and then choosing that experiment from the list under “Headings.” However, Use the Highlighter to your advantage, marking important results, conclusions, concerns, questions for the future, etc.
8. If you are performing a collaborative experiment and information/protocols/etc are in someone else’s digital lab notebook, highlight the relevant location and Insert a Bookmark. They can “Copy Link address” of blue “Link” text of the bookmark and include that in their digital lab notebook. Similarly, if you want to link to someone else’s data, make sure you have a link to their Bookmark!
9. **It is NOT appropriate to just copy & paste from another student’s work**, even if you’re collaborating together - this would be considered plagiarism. Your lab notebook should contain your own writing describing what *you* did: anything that isn’t your writing should be clearly quoted, marked in a different color/style, with a source for the copied text listed. If you copy/paste in a protocol or instructions, you must also list/hyperlink where it came from.
10. You should have **a bound paper lab notebook also**, as for some experiments you can’t have a computer on hand, or you are using printed protocols where you annotate changes/comments as you go, or for signed training forms etc. You can treat this paper copy as a less formal way to keep track of things, where you can scrawl down notes/thoughts as you’re working. **You must always have a date for any material in the paper notebook**, though there’s no other specific structure you need to follow for that. Never use loose paper for experiments - any printouts/etc should be immediately taped or stapled into the notebook. **This digital notebook is your official notebook that will be assessed across the semester**, so all relevant entries and information from your paper notebook should be included in this digital notebook, as a picture of the page (or retyped if needed for clarity).
11. If you have handwritten/printed information from your experiment, it should be in your paper lab notebook with a Date on each page. **YOU MUST REFERENCE THE PAPER NOTEBOOK PAGE NUMBER OF ANYTHING IMPORTANT IN YOUR DIGITAL NOTEBOOK**, though you don’t necessarily need to recopy everything digitally from your paper notebook as long as the page number is clearly marked. It can be helpful to take a quick phone pic of the relevant page and paste it into your digital notebook, rather than retyping/rewriting.
12. If you realize you have misinterpreted data, or that there was a problem with something you previously entered into your notebook, **DO NOT DELETE ANYTHING!** Instead, go to that section and type in your adjusted comment with the date **[YYYY-MM-DD]** and your correction, highlighting the text in **a new color** to make it stand out. If necessary, you can “Strikethrough” previous incorrect text to maintain the record but also make sure it doesn’t mislead you in the future.

# YYYY-MM-DD: Entry Template (Replace w/Experiment Title)

***[Replace the black text below with your entry information!]***

**Purpose**:

What are you trying to do (goals, what you want to accomplish)? WHY is this important (scientific rationale, as well as in the immediate context of this lab)?

**Protocol**:

How will you do the experiment? Step by step procedure summarized here. This must include:

- Hyperlinks to any existing protocol(s) you used
- Full written out protocol(s) that you performed must be included the first time you perform that protocol (hyperlink is NOT ENOUGH the first time!)
- Any adjustments to the protocol must be annotated: use highlighting or font color to indicate changes, and ~~strikethrough~~ to indicate steps that you didn’t perform

**Results**:

This section should include any graphs, images, analytical results, getting across the following:

- What did you observe along the way while you carried out the protocol(s)?
- What do you know/What have you figured out?
- What *don’t* you know yet and still need to figure out in the future?
- If you are performing electrophysiology, this section must include a Cricket Experiment Table, which details every cricket and recording you made, including saved recordings that you don’t end up analyzing or following up with for whatever reason
- If you are performing microscopy, this section must include an Imaging Table, which details everything you took images/videos of, including images of samples that you don’t end up analyzing or pursuing for whatever reason
- This section often includes figures (example microscopy images, screenshots, error messages, phone pictures of experimental setup, etc). Include a figure legend text with each figure
- All digital file names for analysis files must be listed, and hyperlink to them

**Discussion**:

This section overall should address the following:

- What did you end up doing (briefly)? Just a few sentences.
- Have you made any conclusions about your project yet? Are things inconclusive or still in progress?
- What were the challenges in this part of the lab and what could be done to troubleshoot/optimize in the future?
- What are the next steps you need to take?
- What is your plan to find out what you don’t know (problems/questions that came up in the Results)?

# Imaging Table Template

| **File Name**  **(YYMMDD_INIT_Fish_Position_IMG##)** | **Sample Info (genetic BG, age, treatment, etc)** | **Imaging Conditions (microscope, objective, laser settings, framerate, etc)** | **Comments/Observations** |
| --- | --- | --- | --- |
| *Example: 230930_RJEW_F2_P03_IMG03*  ****DELETE THIS ROW**** | *glyT2:gal4; uas:gcamp5g*  *7dpf fish, PTU treated,* | *confocal*  *20x objective*  *30% laser power*  *512x512*  *10 fps* | *Recorded 30s of Spontaneous activity of a cluster of 10 neurons near the left ear. This corresponds to position 03 in the overall fish map (see 230930_RJEW_F2_Overview1 image)*  *A few neurons seemed to be synchronously active!* |
|  |  |  |  |
|  |  |  |  |
|  |  |  |  |

# Cricket Experiment Template

| Experiment Title: | | | Impact of 4-AP on cricket cercal ganglion excitability | | | | |
| --- | --- | --- | --- | --- | --- | --- | --- |
| Date | | | 9/6/23 | | | | |
| Scientist | | | Eric Wengert | | | | |
| Collaborating Scientists | | | Roshan Jain | | | | |
| Acquisition Highpass/Lowpass Filter Settings | | | 160 Hz/3700 Hz; Full volume | | | | |
| Saved File Location | | | This PC/Documents/BYB/2023_09_06 | | | | |
| Cricket ID | Sex | Time of anesthesia | Filename | Time of Recording | Stimulus Description | Treatment Description | Experimental Notes/Observations |
| 01 | M | 2:12 | BYB_Recording_2023-09-06_14.18.00 | 2:18 | 5 seconds of airpuffs at 1 Hz beginning at 5 seconds | Untreated | Cricket began moving part way through the recording leading to some spikes that may not be due to pressure-induced activity in the cercal ganglion. |
| 01 | M | 2:12 | BYB_Recording_2023-09-06_14.21.02 | 2:21 | 5 seconds of airpuffs at 1 Hz beginning at 5 seconds | 10 uL of 0.1 M 4-AP in H_2_O injected 30 seconds before start of recording. | Clear increase in spiking amplitude and frequency, however, baseline became more noisy after the injection of 4-AP. |
| 02 | F | 2:30 |  |  |  |  |  |
|  |  |  |  |  |  |  |  |

(be sure to delete example entries and replace them with your actual experimental info!)

# Fall Lab Safety Form

[Insert your signed and dated safety form here]
